# 3D Localization for Light-Field Microscopy via Convolutional Sparse Coding on Epipolar Images

**DOI:** 10.1109/TCI.2020.2997301

**Published:** 2020-05-29

**Authors:** Pingfan Song, Herman Verinaz Jadan, Carmel L. Howe, Peter Quicke, Amanda J. Foust, Pier Luigi Dragotti

**Affiliations:** 1 Department of Electronic & Electrical EngineeringImperial College London 4615 London SW7 2AZ U.K.; 2 Department of Bioengineering, and Center for NeurotechnologyImperial College London 4615 London SW7 2AZ U.K.

**Keywords:** Light-field microscopy, epi-polar plane image, convolutional sparse coding, depth-aware dictionary

## Abstract

Light-field microscopy (LFM) is a type of all-optical imaging system that is able to capture 4D geometric information of light rays and can reconstruct a 3D model from a single snapshot. In this paper, we propose a new 3D localization approach to effectively detect 3D positions of neuronal cells from a single light-field image with high accuracy and outstanding robustness to light scattering. This is achieved by constructing a depth-aware dictionary and by combining it with convolutional sparse coding. Specifically, our approach includes 3 key parts: light-field calibration, depth-aware dictionary construction, and localization based on convolutional sparse coding (CSC). In the first part, an observed raw light-field image is calibrated and then decoded into a two-plane parameterized 4D format which leads to the epi-polar plane image (EPI). The second part involves simulating a set of light-fields using a wave-optics forward model for a ball-shaped volume that is located at different depths. Then, a depth-aware dictionary is constructed where each element is a synthetic EPI associated to a specific depth. Finally, by taking full advantage of the sparsity prior and shift-invariance property of EPI, 3D localization is achieved via convolutional sparse coding on an observed EPI with respect to the depth-aware EPI dictionary. We evaluate our approach on both non-scattering specimen (fluorescent beads suspended in agarose gel) and scattering media (brain tissues of genetically encoded mice). Extensive experiments demonstrate that our approach can reliably detect the 3D positions of granular targets with small Root Mean Square Error (RMSE), high robustness to optical aberration and light scattering in mammalian brain tissues.

## I. Introduction

Understanding mechanisms of perception, cognition, and complex behavior emerging from global dynamics of neuronal network activity is a fundamental problem in neuroscience. Progress depends on development of technologies to simultaneously track the activity of hundreds to thousands of neurons. Optical technologies could achieve this by imaging photons from many neurons in parallel without mechanically perturbing the brain tissue. In particular, dyes and proteins have been engineered to transduce changes in membrane potential and calcium concentration into optical contrasts such as fluorescence [1], [2]. Despite its immense promise, optically imaging the activity of mammalian neuronal networks poses two key challenges: first, neurons are distributed in three spatial dimensions while traditional microscopes focus on a single two-dimensional plane; and second, mammalian brain is highly scattering. Not only absorbing light, mammalian brain tissues also scatter incident light many times, causing images to look diffused. Imaging through scattering tissue remains an important problem in optics, and advanced methods are required.

Two-photon microscopy [3]–[6] is one of the most popular imaging techniques due to several significant advantages on deeper tissue penetration, efficient light detection, reduced photo-bleaching, and mitigating the scattering issue. These benefits come from exploitation of near-infrared (longer wave-length) light for reducing scattering and absorption, as well as exploitation of the non-linear excitation property of multiphoton absorption to restrict fluorescence excitation to a small local spot. Such localized excitation has been applied to point by point scanning when imaging a 3D volume. However, this serial acquisition limits the imaging speed. Efforts to increase acquisition speed include engineered beam trajectories [7]–[10], spatial and/or temporal multiplexing of multiple foci [6], [11]–[17], as well as sculpting fluoroscence excitation into an extended point-spread function [18]–[21], either scanned or targeted statically onto neurons of interest.

By leveraging light-field imaging [22], light-field microscopy (LFM) [23]–[28] provides an alternative for 3D imaging of neural activity with fast frame rate. In contrast to conventional optical microscopy that records only lateral information as a 2D projection of light rays, LFM is effectively a 3D optical imaging technique with the capability of simultaneously gathering both position and angular information of the incident light rays arriving at the sensor. This is achieved by incorporating a microlens array (MLA) at the original imaging plane and by moving the imaging sensor to the rear focal plane of the microlenses [23]. With this structure, LFM is able to acquire 4D data containing both spatial positions and direction of propagation of light rays with a single snapshot. This non-scanning imaging mechanism contributes to high light efficiency and fast imaging speed, facilitating the recording of neural population activity at high frame rates [26]–[28]. The promising application at the tissue level holds great potential for observing structures and dynamics across whole brain volumes.

However, the benefits of light-field imaging on light efficiency and imaging speed come at the cost of reduced spatial resolution due to the recording of angular information using some pixels. It also suffers from substantial image degradation due to scattering in deep layers of brain tissue. To this end, various approaches were developed to improve spatial resolution via, for example, 3D deconvolution [25], [26], sparse decomposition in phase-space [28]–[30].

**Fig. 1.**
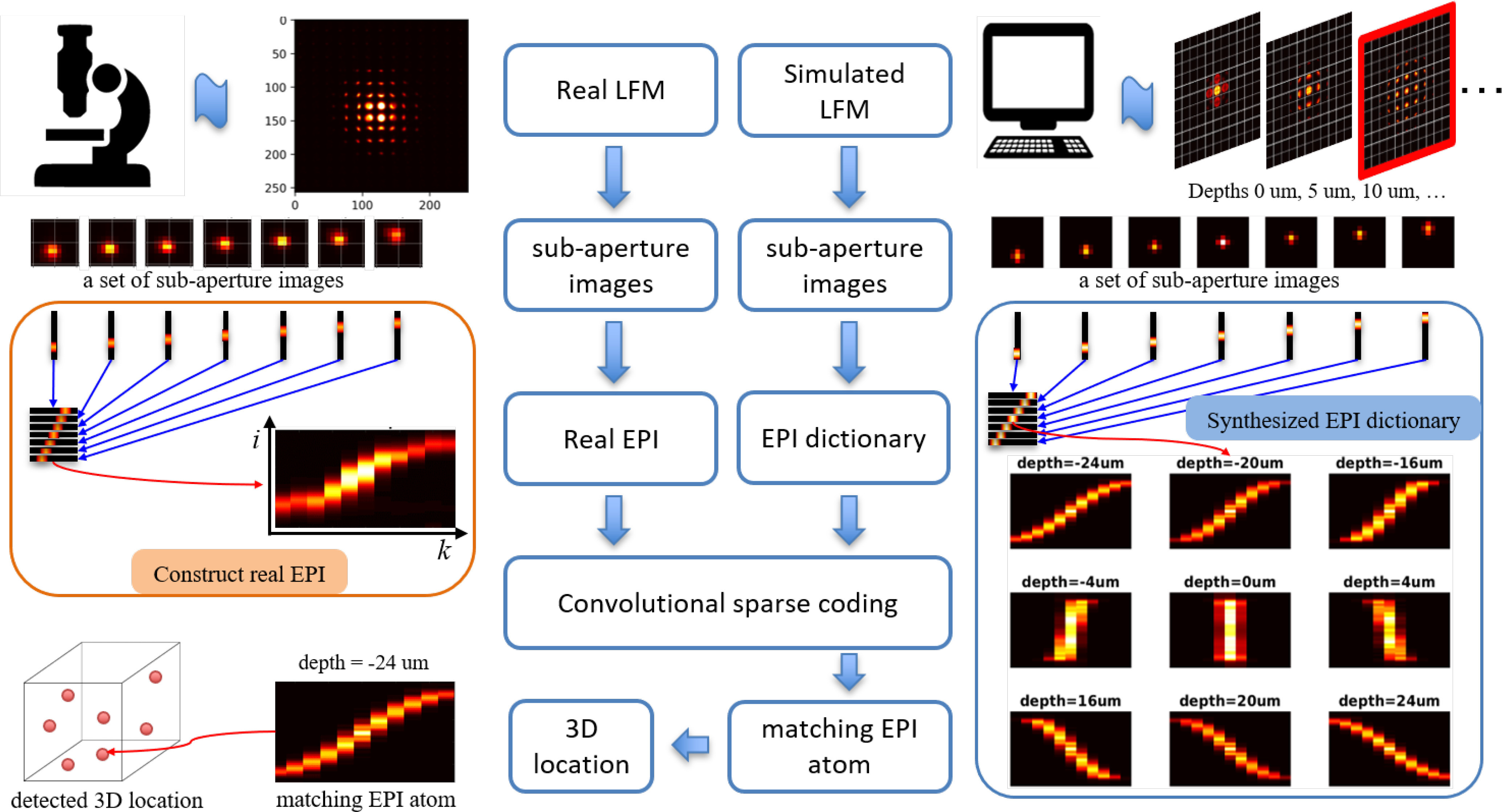
Flow chart for localization using convolutional sparse coding (CSC) on epipolar plane images. The real (on the left-hand side) and simulated (on the right-hand side) light-field microscopy (LFM) images are first calibrated and decoded into two-plane parameterized 4D format in order to obtain sub-aperture images. Then, the epipolar plane images and dictionary are constructed from the sub-aperture images. Finally, convolutional sparse coding is performed on the EPI with respect to the EPI dictionary to detect the 3D location of targets.
• Considering that neurons have a compact somata, they are effectively modeled as ball-shaped volumetric sources. A wave-optics forward model [24] is adopted to synthesize a series of light-field images for a ball-shaped volume located at different depths.
• From the set of synthesized light-field images, a novel *depth-aware* dictionary is constructed, in which each element, often called atom, is an EPI associated with a specific depth. This EPI dictionary serves as the bridge to link an observed EPI to the 3D positions of the targets via our localization algorithm.
• By exploiting spatial sparsity and shift-invariance properties of EPI, we develop a specific convolutional sparse coding (CSC) algorithm for 3D localization from a single light-field image.

Different from existing methods, we propose a new approach to address the issues in LFM imaging and provide the ability to measure 3D positions of neurons from a single snapshot with high accuracy, efficiency and robustness. Our approach is based on the epi-polar plane image (EPI), an effective tool to analyze 3D information in 4D light-field data [31]–[35]. Since each point source traces out a tilted line in an EPI, the intrinsic dimension of an EPI is much lower than the ambient dimension of the raw light-field data, making the 3D localization highly tractable and thereby offering a path toward efficient 3D localization. Moreover, by skipping the time-consuming and error-prone 3D volume image reconstruction explicitly, our approach reduces computational complexity significantly and improves localization accuracy. The overall procedures of the proposed approach are shown in Fig. 1. The novelties of our approach include the following aspects:

• An automatic calibration and decoding method is developed to convert a raw 2D light-field image to the two-plane parameterized 4D format, which allows the EPI to be built accurately.

## II. Background

**Fig. 2.**
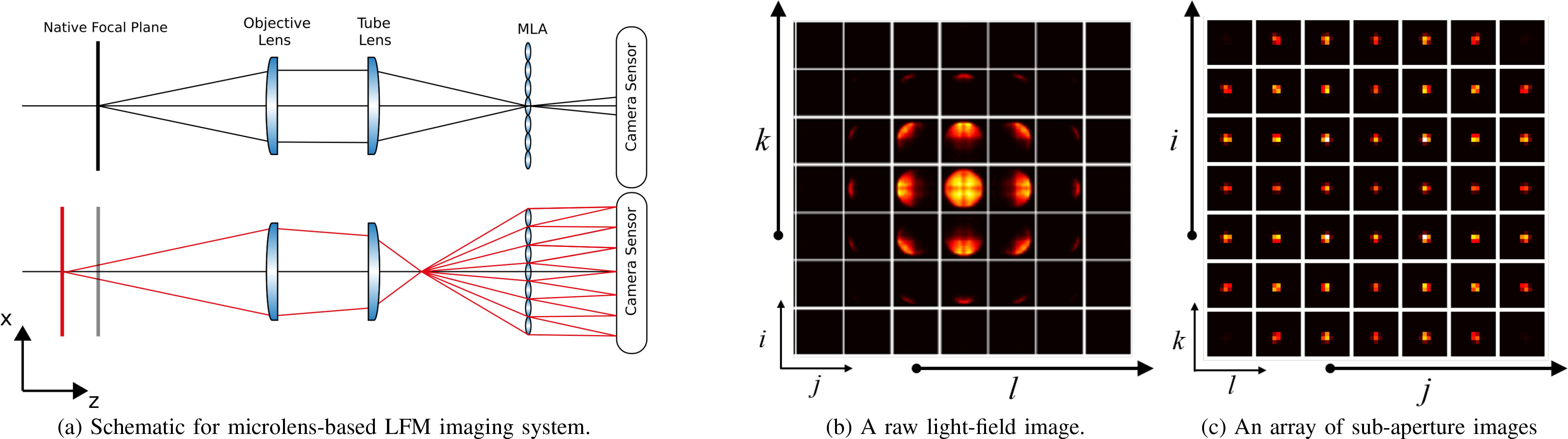
Illustration of microlens-based light-field imaging. (a) Schematic for microlens-based LFM imaging system. MLA: microlens array. (b) A raw 2D light-field image $I(i,j,k,l)$ of a bead with 10 $\mu$m diameter at a certain depth. Simulated for a microlens-based light-field system. White lines indicate the virtual profile of lenslets at the imaging side of the system, and each square represents a micro-image associated with a specific lenslet. (c) An array of sub-aperture images $I_{4D}(i,j,k,l)$, a.k.a. multi-view images, are converted from the raw light-field image. Each sub-aperture image indicates a specific view specified by $(i,j)$. Namely, it is composed of pixels that share the same relative position $(i,j)$ in each micro-image.

**Light-field imaging and two-plane parameterization:** Our LFM system adopts a MLA based optical design [23]. The schematic diagram is shown in Fig. 2(a), where a MLA is inserted at the imaging plane between a 4-f optical system (consisting of an objective lens and tube lens) and the camera sensor. Similar designs have also been adopted in commercial light-field cameras via inserting a MLA between the main lens and sensor, such as Lytro Illum by Lytro Inc. [36], [37] and Raytrix GmbH [38].

According to ray-optics, each lenslet in the MLA is treated as an ideal pinhole and the main lens is treated as a thin lens. Thus, Fig. 2(a) indicates that the coordinates $(k,l)$ of the lenslets and the coordinates $(i,j)$ of the pixels behind each corresponding lenslet lead to a radiance-valued function $I(i,j,k,l)$ which determines each ray uniquely by the quadruple $(i,j,k,l)$ and assigned radiance value $I$. In other words, $(k,l)$ index the spatial positions of lenslets while $(i,j)$ index the relative positions of pixels behind each corresponding lenslet. Namely, each pixel behind a lenslet captures a specific perspective.

The microlens-based light-field imaging systems aim to transform the light-field from the world space into the image space of the main lens and thereby sampling the light-field at the sensor plane. Each lenslet with its underlying group of pixels forms an in-camera sampling scheme, analogous to a tiny camera with very few pixels, that observes the in-camera light-field. The observation recorded by all the pixels in a sensor leads to a raw light-field image $I(i,j,k,l)$, as shown in Fig. 2(b), where each square represents a micro-image associated with a specific lenslet (7 by 7 micro-images are shown here with white lines indicating the virtual profile of lenslets at the imaging side of the system). Note that exact coordinates $(i,j,k,l)$ remains unknown in the raw light-field image until the profile of lenslets is computed. That is why it is called a 2D raw light-field image even though it already contains 4D information.

Once the profile of lenslets is computed during calibration, fixing $i$ and $j$ leads to an image $I(i,j,:,:)$, referred to as a sub-aperture image, that is composed of pixels that share the same relative position $(i,j)$ in each micro-image, thus indicating a specific view specified by $(i,j)$. An array of sub-aperture images $I_{4D}(i,j,k,l)$, as shown in Fig. 2(c), are obtained from the raw light-field image in Fig. 2(b) by rearranging the pixels referring to their angular positions $(i,j)$. It is noticed that the perspective changes along rows (from left to right) and columns (from up to down). Such sub-aperture images depict varying perspectives of the scene, which is similar to the multi-view images captured by a camera-array. This confirms that the microlens-based light-field imaging system allows for multi-view acquisition. Therefore, using sub-aperture images as a bridge, the representation for microlens-based light-field can be converted to an equivalent representation for camera-array based light-field which is often parameterized by two parallel planes.

**Fig. 3.**
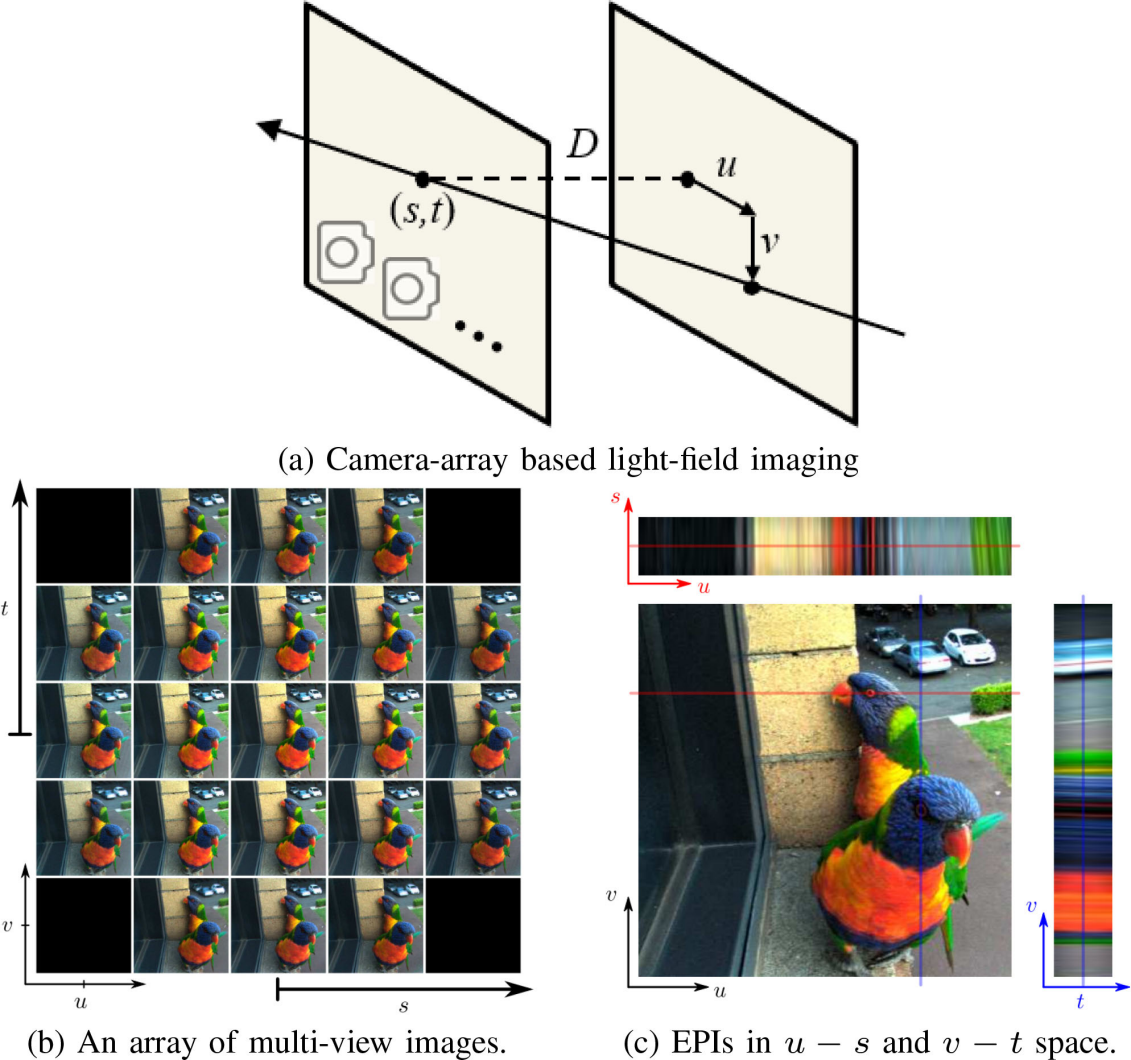
Illustration of camera-array based light-field imaging. (a) For a camera-array based light-field system, a light ray that propagates from the surface of the scene is uniquely determined by the intersections with two parallel planes, leading to a relative two-plane parametrization of light-field. By convention, the $s-t$ plane is closer to the camera, and the $u-v$ plane is closer to the scene. (b) An array of multi-view images shows that the view changes along different directions. (c) A EPI (up) in $u-s$ space for fixed $(v,t)$ and an EPI (right) in $v-t$ space for fixed $(u,s)$. (Images are from  [39], [40].)

Specifically, the light-field captured by a camera-array is commonly represented by *relative* two-plane parameterization, as shown in Fig. 3(a), where a light ray that propagates from the surface of the scene is determined by the intersections with two parallel planes. Following the notations in the Lumigraph paper [33], the parameterization then consists of the intersection position $(u,
                        v)$ of the ray with the first plane (closer to the scene and called the image plane), and the intersection position $(s,
                    t)$ with the other parallel plane (closer to the camera and called the camera plane) at a distance $D$. Note, the intersection $(u,
                    v)$ denotes the relative position with respect to the intersection $(s,
                    t)$, which accounts for the “*relative*” two-plane parameterization. By convention, $D$ is often set to be the focal length $f$. In this way, the light-field is represented by a radiance-valued function $I(u,v,s,t)$ which determines each ray uniquely by the quadruple $(u,v,s,t)$ and assigned radiance value $I$. A 2D signal obtained by fixing $s$ and $t$ resembles an image with a specific perspective, whereas fixed values of $u$ and $v$ give a hypothetical radiance function. Fig. 3(b) shows an array of multi-view images that are tiled together according to their $(s,t)$ positions to visualize the 4D light-field. It can be noted that under the two-plane parameterization, the two types of light-field systems are analogous, with $(i,j)$ equivalent to $(s,t)$ and $(k,l)$ equivalent to $(u,v)$.

**Fig. 4.**
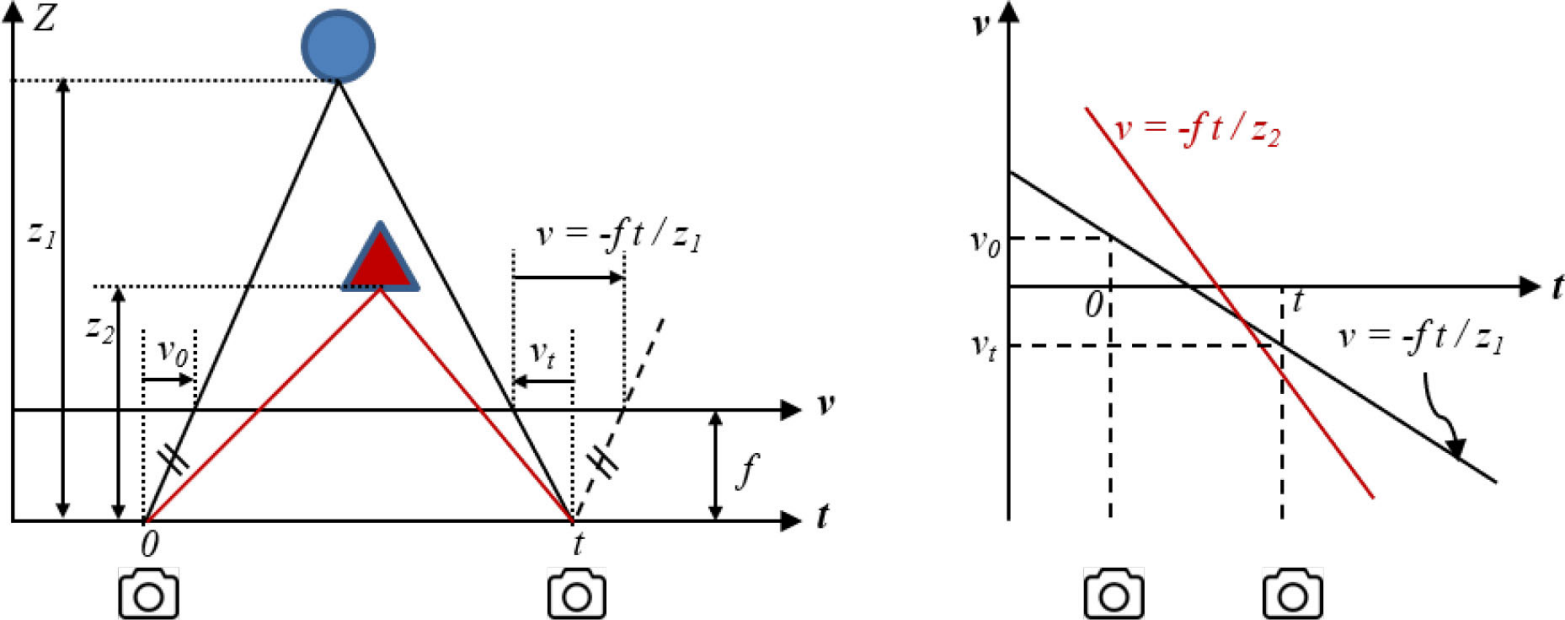
An illustration of 2D light-field (with fixed $s$ and $u$) and EPI. Left: each point is observed by two cameras with centers at 0 and t; Right: Stacking pixels captured along the camera path leads to an epipolar line in the EPI for the corresponding point. The slope is related to the depth of the point in world space. The deeper the point, the more tilted the epipolar line.

**Fig. 5.**
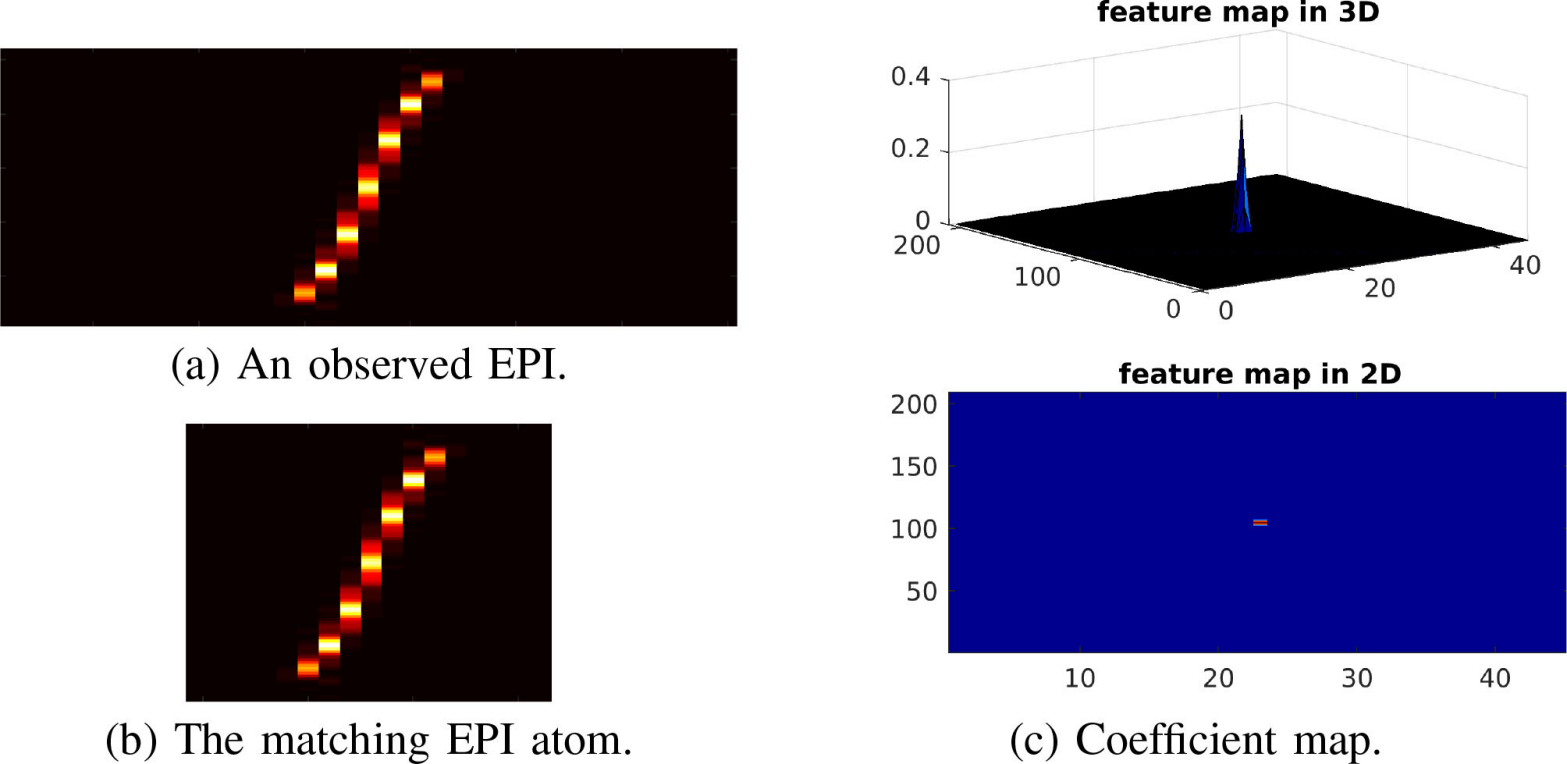
Illustration of pattern recognition using convolutional sparse coding on EPI. (a) An observed EPI constructed from a raw light-field image for a ball-shaped volume at 10 $\mu {\text m}$ depth. It contains an epipolar line corresponding to the ball-shaped volume. (b) The matching EPI atom in a simulated EPI dictionary. (c) Convolving the EPI with the matching atom results in a coefficient map with a peak at the best overlapping position.

**Epipolar plane image:** Fixing $s$ and $u$ (or $t$ and $v$) gives rise to a 2D slice with angular and spatial directions, referred to as an Epipolar Plane Image (EPI) [31]–[35], as shown in Fig. 3(c). A point in the world space traces out a straight and tilted line, referred to as an epipolar line, determined by only a few meaningful parameters, as shown in Fig. 4. In particular, the slope is inversely proportional to the depth while the horizontal position is proportional to the lateral positions of the point in the real world scene. The EPI allows an easy illustration of the light-field in two dimensions and its characteristics lay the foundation for our study and inspire us to develop effective algorithms for 3D localization. Similarly, fixing $i$ and $k$ (resp. $j$ and $l$) gives rise to an EPI $I(i,:,k,:)$ (resp. $I(:,j,:,l)$) in which the two axes represent spatial and angular dimension, and each epipolar line reveals the depth and lateral positions for the corresponding point in the scene, as shown in Fig. 5(a).

## III. Dictionary Construction

### A. Preliminaries

The proposed 3D localization approach is based on convolutional sparse coding for EPIs. Our approach comes from the following insights.

**Depth related property of EPI:** As introduced in the background, a raw light-field image captured by a microlens-based light-field imaging system can be converted into an array of multi-view images with two-plane parameterized 4D format. Then, EPIs can be constructed from the 4D data, in which a point forms an epipolar line, as shown in Fig. 4. A change in the depth position results in the change of the slope of the straight line in the EPI, that is, the shift operation in the depth axis corresponds to a shearing operation in the EPI domain. Specifically, the deeper the point source, the larger the slope of the line, as shown in Fig. 4. This characteristic of EPI inspires us to leverage EPI as an effective feature to perform pattern recognition in order to detect the depths of target objects.

**Shift-invariance property of EPI:** Given the fixed depth of a point source, shifting its lateral position along a spatial dimension, e.g. horizontal direction, results in the shift of the epipolar line in the EPI along corresponding spatial dimension. Specifically, the horizontal (resp. vertical) shift of a point source corresponds to the shift of the epipolar line along the spatial dimension in the horizontal (resp. vertical) EPI. Such translation-invariance property accounts for why convolution is an effective operation to search for specific patterns and to perform pattern recognition. Relying on this insight, we develop an algorithm to efficiently search and identify target EPI patterns in a sub-space spanned by a set of elements in an EPI dictionary. In such an EPI dictionary, each element is an EPI associated with a specific depth, thus it allows shearing and shift being taken into account through convolution. Furthermore, the size of each EPI atom can be much smaller than the input observed EPI, and thus significantly reducing computational complexity.

**Ray-optics model vs wave-optics model:** A ray-optics forward model is commonly used to formulate the light-field imaging process for opaque scenes and diffusely reflecting objects that are at typical macroscopic photographic scales. However, for LFM, the samples are so small that they are largely transparent or semi-transparent and the diffraction effects of light need to be taken into account. To this end, a wave-optics model should be used to replace the rays-optics model to better formulate the imaging process. Here, we exploit a wave-optics forward model introduced in [24] to emulate the imaging process which has been proved to be more accurate than the ray-optics model. In this way, we ensure that the simulated light-field images match real images. Consequently, EPIs constructed from the simulated light-field data closely match those obtained from real data.

Based on above insights and analysis, it is observed that the convolution of an EPI image with a matching EPI atom gives a spiking coefficient map with the highest response at the overlapping position, as shown in Fig. 5. In contrast, convolution with a non-matching EPI (that is an EPI related to a different depth) leads to an unstructured, lower response at the overlapping position in the coefficient map. If the EPI is sparse, that is, contains only a few epipolar lines, the coefficient maps are also sparse with only a few large responses. These account for why convolutional sparse coding on EPIs can identify the matching atoms, thereby leading to the depth position detection via searching a look-up-table. Accordingly, it also allows identifying the lateral positions from the coefficient maps by examining the largest responses.

### B. Wave-Optics Model for Synthesizing EPI Dictionary

Our localization approach requires an EPI dictionary that contains a set of EPIs corresponding to different depths. We propose simulating LFM imaging and synthesizing such a dictionary by exploiting a wave-optics forward model [24].

The wave-optics forward model describes how to evaluate the light-field for an ideal point source that passes through a LFM system, i.e. the impulse response function, a.k.a. point spread function (PSF) which characterizes the properties of the optical system.

In particular, given an ideal point source located at $\mathbf {p}=(p_1, p_2,
                        p_3)$, the PSF $h(\mathbf {x}, \mathbf
                        {p})$ at the sensor plane is given by

\begin{equation*}
                            h(\mathbf {x},\mathbf {p}) = \mathcal {F}^{-1} \lbrace \mathcal {F}
                            \lbrace U_i(\mathbf {x},\mathbf {p}) \Phi (\mathbf {x}) \rbrace
                            G(\hat{\mathbf {x}}) \rbrace \tag{1} \end{equation*}

where, $U_i(\mathbf {x},\mathbf {p}) = U_o(-\mathbf {x}/M,\mathbf
                            {p})$ and $U_o$ denotes the virtual wavefront at the native object plane computed using Debye theory. $U_i$ is the resulting light-field at the native image plane of a 4-f system and is formulated as the inverted and stretched version of $U_o(\mathbf
                            {x},\mathbf {p})$. $\Phi (\mathbf {x})$ denotes the lens mask of a MLA that is described as the convolution of a 2D Dirac impulse with the transmittance of a lenslet. After multiplying $U_i(\mathbf {x},\mathbf
                            {p})$ by the lens mask $\Phi (\mathbf
                            {x})$, the propagation of the result from the MLA to the sensor plane using the paraxial approximation can be formulated using the transfer function $G(\hat{\mathbf {x}}) = \exp (-\frac{i}{4\pi } \lambda f_{ML} \Vert
                            \hat{\mathbf {x}}\Vert _2^2))$ where $f_{ML}$ denotes the focal length of the MLA. More details can be found in the supplemental material VII-A or literature [24].

Given the PSF, the wavefront recorded at the sensor plane is described using a general linear superposition integral [24]:

\begin{equation*} f(\mathbf {x}) = \int |h(\mathbf {x},\mathbf {p})|^2
                            g(\mathbf {p}) d \mathbf {p}, \tag{2} \end{equation*}

where $\mathbf {p}\in \mathcal {R}^3$ is the position in a volume containing isotropic emitters whose combined intensities are distributed according to $g(\mathbf {p})$.

**Fig. 6.**
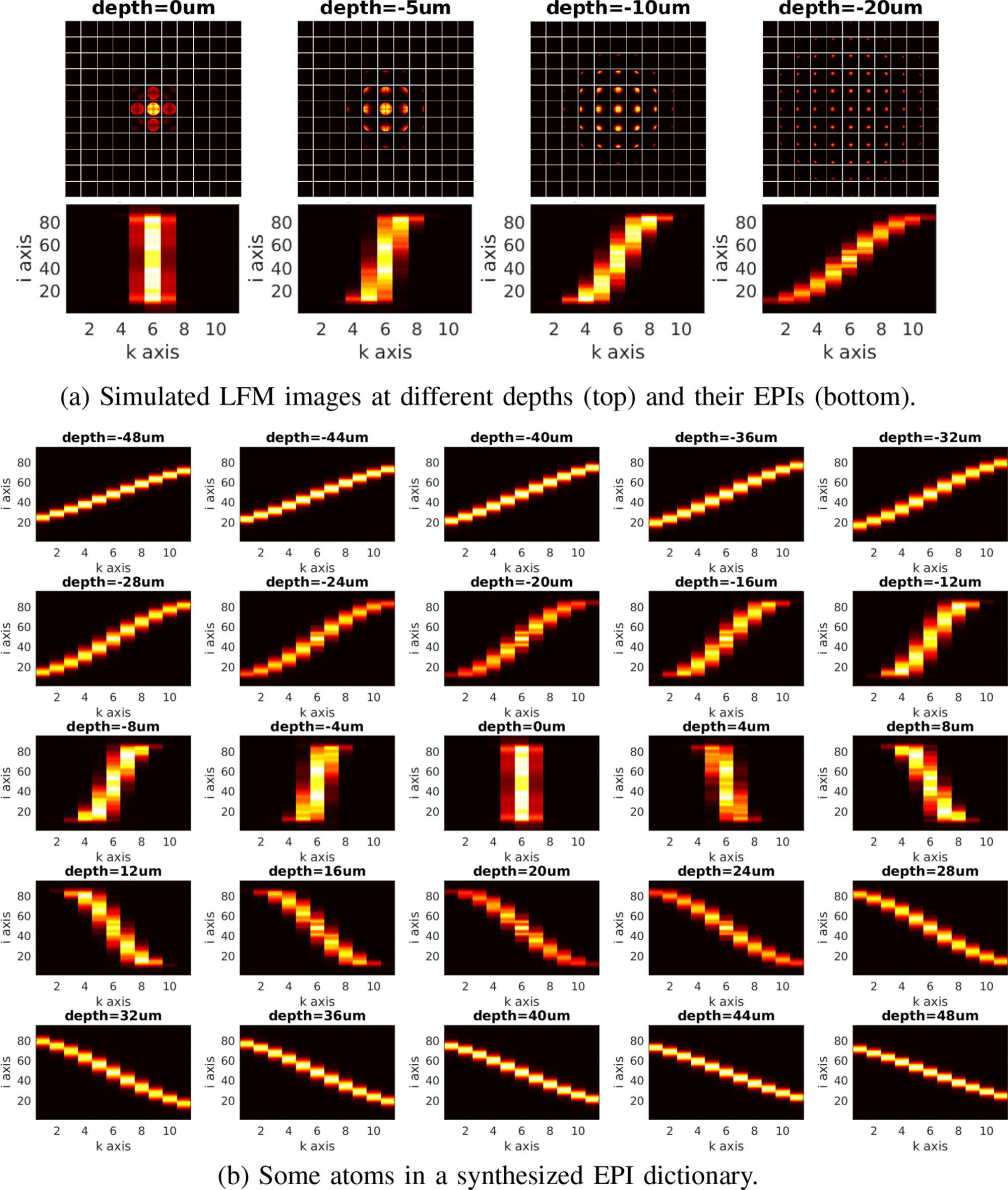
Simulated EPI dictionary. (a) Some examples for simulated LFM images for a volume ball of diameter 10 $\mu {\text m}$ at different depths and corresponding EPIs. (b) Some atoms in the simulated EPI dictionary, indicating that the slope in an EPI is associated with the depth of the volume ball. Here, the horizontal and vertical EPIs constructed from simulated LFM images are the same to each other.

Observing that compact somata of neuronal cells results in fluorescence in the cytoplasm mainly confined to a tiny (around 10 $\mu {\text m}$ diameter) region, it is therefore reasonable to model a neuron as a ball-shaped volume of 10 $\mu {\text
                            m}$ diameter. Accordingly, we simulate a series of light-field images for such a ball-shaped volume located at different depths and then construct EPIs from them to synthesize a depth-aware EPI dictionary. Specifically, given the ball volume at a specific depth, we discretize it into points on a regular grid, and use the aforementioned wave-optics forward model and the general linear superposition integral operation to produce a synthetic light-field image for this volume. We then convert the light-field into the standard 4D format according to the two-plane parameterization, and construct an EPI associated with the specified depth. In this way, a series of EPIs associated with a variety of depths are generated and form a depth-aware EPI dictionary, as shown in Fig. 6.

We note that due to the adopted wave-optics model, the lines in the EPIs are not straight but slightly curved due to shearing. Nevertheless, the introduced characteristic and translation-invariance property are still valid. Therefore, the convolution based pattern recognition still makes sense and can be adapted for the specific application.

## IV. Localization Algorithm

**Fig. 7.**
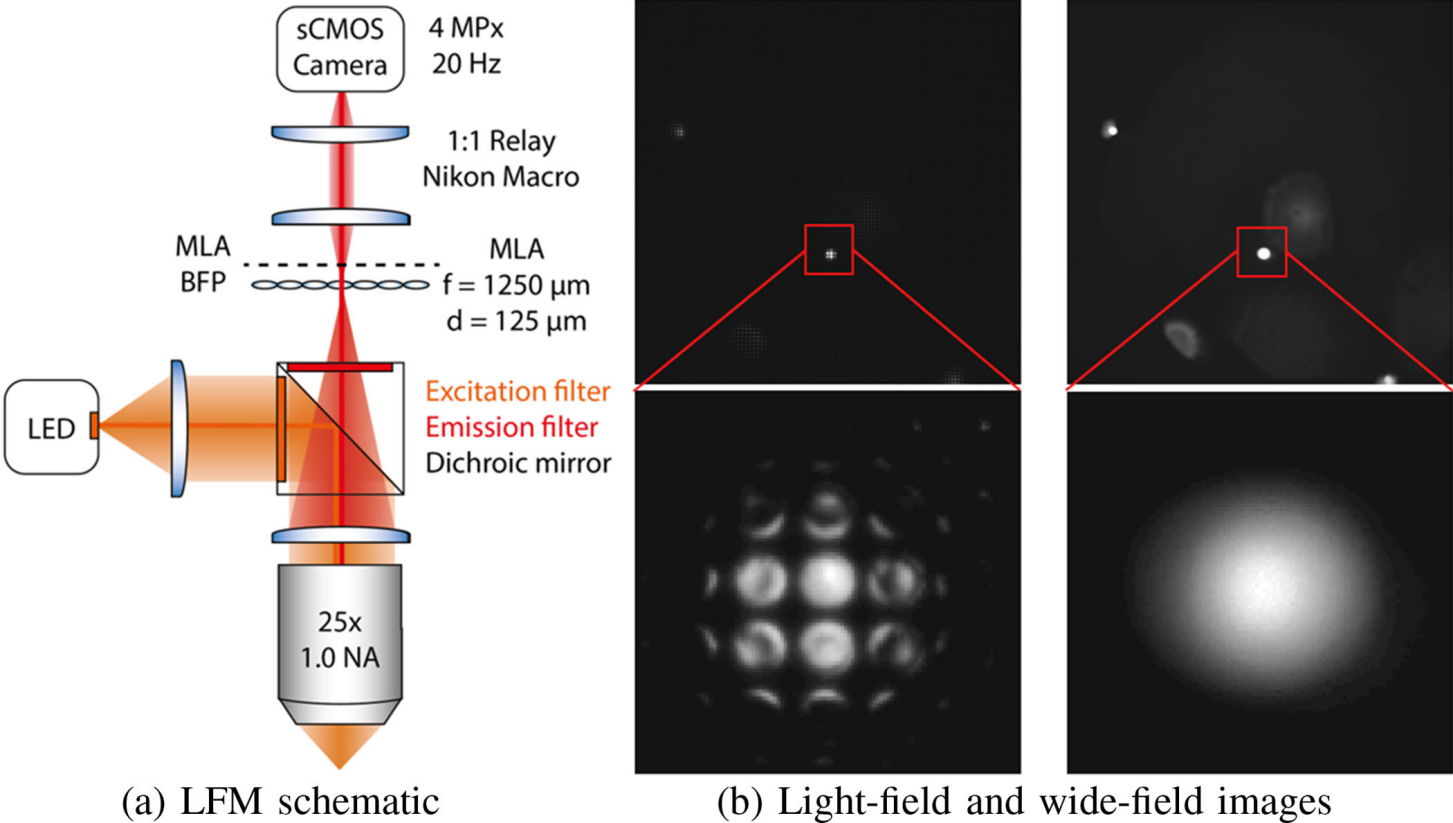
(a) LFM schematic. The designed LFM is modified from a fluorescence microscopy by inserting a MLA at the imaging plane of an objective lens and tube lens with a CMOS sensor placed at its back focal plane. (b) Comparing light-field (left) and wide-field (right) images for a fluorescent bead of 10 $\mu {\text
                                m}$ diameter. The zoom-in region shows that light-field image is composed of small round spots which correspond to the back-aperture of each lenslet. The wide-field image was taken using the same microscope with the MLA removed.

Based on the designed depth-aware EPI dictionary, we are now in a position to describe the proposed location algorithm. We first note that the real data used in our experiments are provided using a light-field microscope designed in our laboratory. As shown in Fig. 7, the microscope is modified from a fluorescence microscopy by inserting a MLA (pitch 125 $\mu {\text
                        m}$, f/10, RPC Photonics) at the imaging plane of an objective lens ($25\times$, $NA=1.0$, Olympus) and tube lens (180 nm, Thorlabs) with a CMOS sensor (ORCA Flash 4, Hamamatsu) placed at its back focal plane. By the principles of light-field imaging, each lenslet records the angular distribution of light rays, therefore such design allows to capture both position and direction of propagation of light rays with a single-shot in a 2D intensity image. We refer to the supplemental material (Subsection VII-B) for a description of the specifics of our microscope.

The location algorithm operates in two steps. We first need to calibrate the LFM. Contrary to standard approaches that perform calibration off-line and require a white image, we achieve this using an out-of-focus real light-field image of the experiment. This is described in Section IV(A). In Section IV(B), we then describe our localization approach based on convolutional sparse coding.

### A. Calibration and EPI Construction

In this part, we introduce the procedures for calibrating the raw LFM images obtained from our LFM system, as well as constructing sub-aperture images and EPI images. Note that, our approach differs from conventional light-field decoding and calibration performed with white images as we use a raw, out-of-focus light-field image. This ensures a better matching of the detected parameters with the target data, as well as simplifies the whole procedure by eliminating the demand for acquisition of white images which are usually harder to obtain.

#### 1) Detection of Rotation Angle and Lenslet Pitch

Since a raw LFM image may be rotated, we need to compute the rotation angle in order to perform rotation calibration, followed by the detection of the lenslet pitch.

**Fig. 8.**
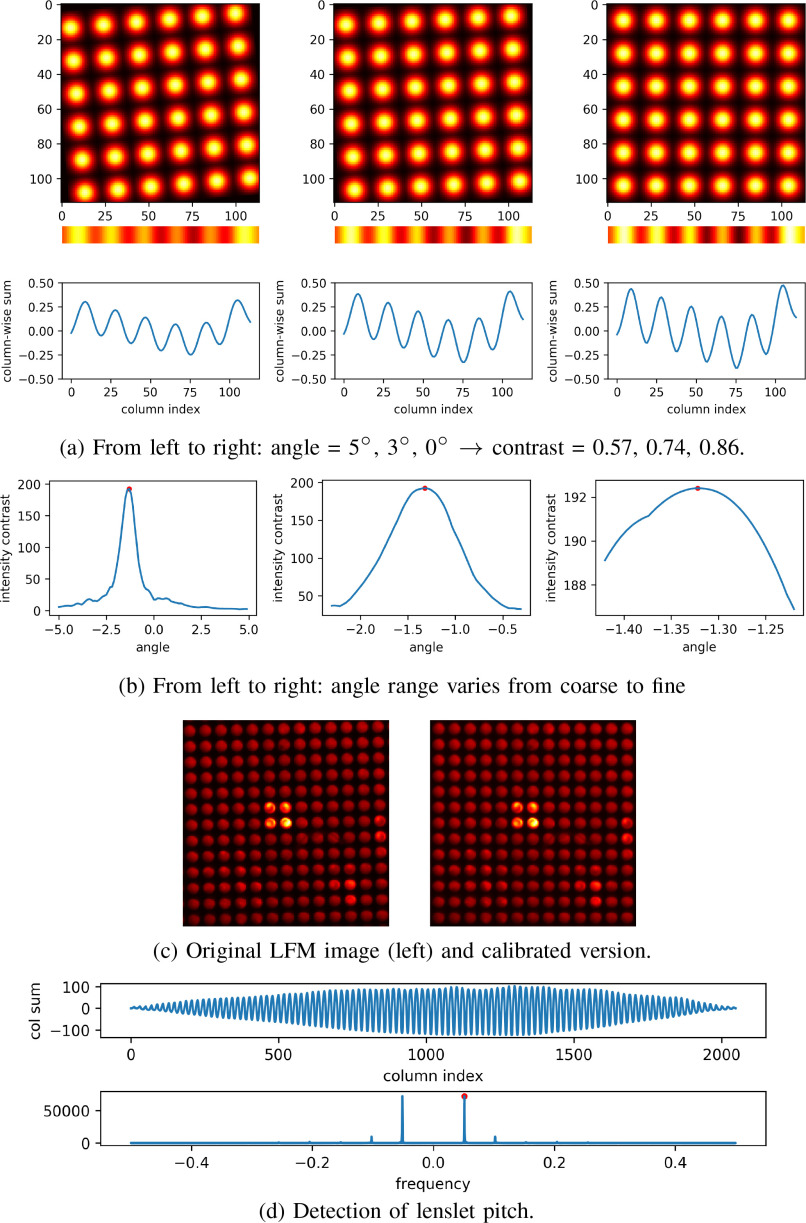
Automatic angle and pitch detection for calibration. (a) illustration of the relation between the intensity contrast and the rotation angle. (b) angle detection via computing the intensity contrast at different angles. (c) an example of rotation calibration using detected angle. (d) Detection of lenslet pitch in frequency domain.

Our angle detection approach is based on the observation that a light-field image shows the grid structure of the MLA with bright and dark spots. After performing column-wise summation, the resulting row vector looks like a stripe with varying levels of brightness. We define the intensity contrast by the difference between the maximum and minimum intensity in the stripe. It is clear that the intensity contrast depends on the rotation angle of the image. Specifically, the smaller the rotation angle, the higher the intensity contrast, as shown in Fig. 8(a) for the case of angle 5$^\circ$, 3$^\circ$, and 0$^\circ$. Taking the extreme case for example, when the rotation angle is zero, all the brightest pixels are added together and the same operation is applied to the darkest pixels, therefore the difference between the maximum and minimum intensity reaches the highest level.

By exploiting this fact, we develop a coarse-to-fine approach to detect the rotation angle progressively. We first search the angle in a coarse range, e.g. $[-5^\circ, 5^\circ
                                ]$ with an increment step of $0.1^\circ$. In each step, the whole image is rotated by the increment step and then pixels are added along the columns, leading to a row vector. After applying a Butterworth highpass filter on the row vector to remove the DC component, the intensity contrast of the filtered vector is computed for the current rotation angle. In this way, we obtain an intensity contrast curve with respect to a series of rotation angles, as shown in Fig. 8(b). The rotation angle corresponding to the highest intensity contrast will be the detection result. Then, we further refine the detection result in a smaller angle range, e.g. $[\alpha -1^\circ, \alpha +1^\circ
                            ]$ where $\alpha$ is the detected angle. One example of rotation calibration using the detected angle is shown in Fig. 8(c). It demonstrates that the raw LFM image can be accurately rotated back to the zero-angle position using the proposed method.

Given the rotation calibration result, the detection of lenslet pitch is performed in the frequency domain, as shown in Fig. 8(d). The idea is that if we add the pixels of the calibrated LFM image along the columns, the resulting 1D signal is approximately periodic and the period corresponds to the pitch of the lenslets. Therefore, a fast Fourier transform (FFT) is performed on the signal to find the largest frequency which represents the changing rate of the intensity. Accordingly, the reciprocal of the largest frequency gives the period of the signal which approximates the lenslet pitch.

Algorithm 1: **Center Detection for MLA.**
**Input:**
  Raw light-field image. Lenslet pitch. A proper threshold for binarization.
**Output:**
  A location map for the MLA centers.
**Procedures:**
1) Design a disc-shape kernel with the diameter of the disc equal to the lenslet pitch. Alternatively, for better robustness, we design a multi-disc-shape kernel that consists of $n \times n$ (e.g. $3 \times
                                        3$) identical discs, with the diameter of each disc equal to the lenslet pitch.
2) Binarize the LFM image with a manually specified threshold.
3) Use a window to extract a region of interest (ROI) from the binarized LFM image and then perform convolution on the ROI using the designed kernel.
4) The point with the largest value in the convolved image is found to serve as the center of the ROI.
5) Move the window by a pitch. Repeat the procedure (3)–(5) until all the centers in the binarized LFM image are detected.
6) Perform average along each row and each column to give the final results.

#### 2) Center Detection for MLA

Center detection and calibration for a MLA is an imperative operation that facilitates accurate decoding of raw LFM data into the standard 4D format. To this end, we introduce a robust center detection method that is able to take advantage of neighbourhood information during the detection.

**Fig. 9.**
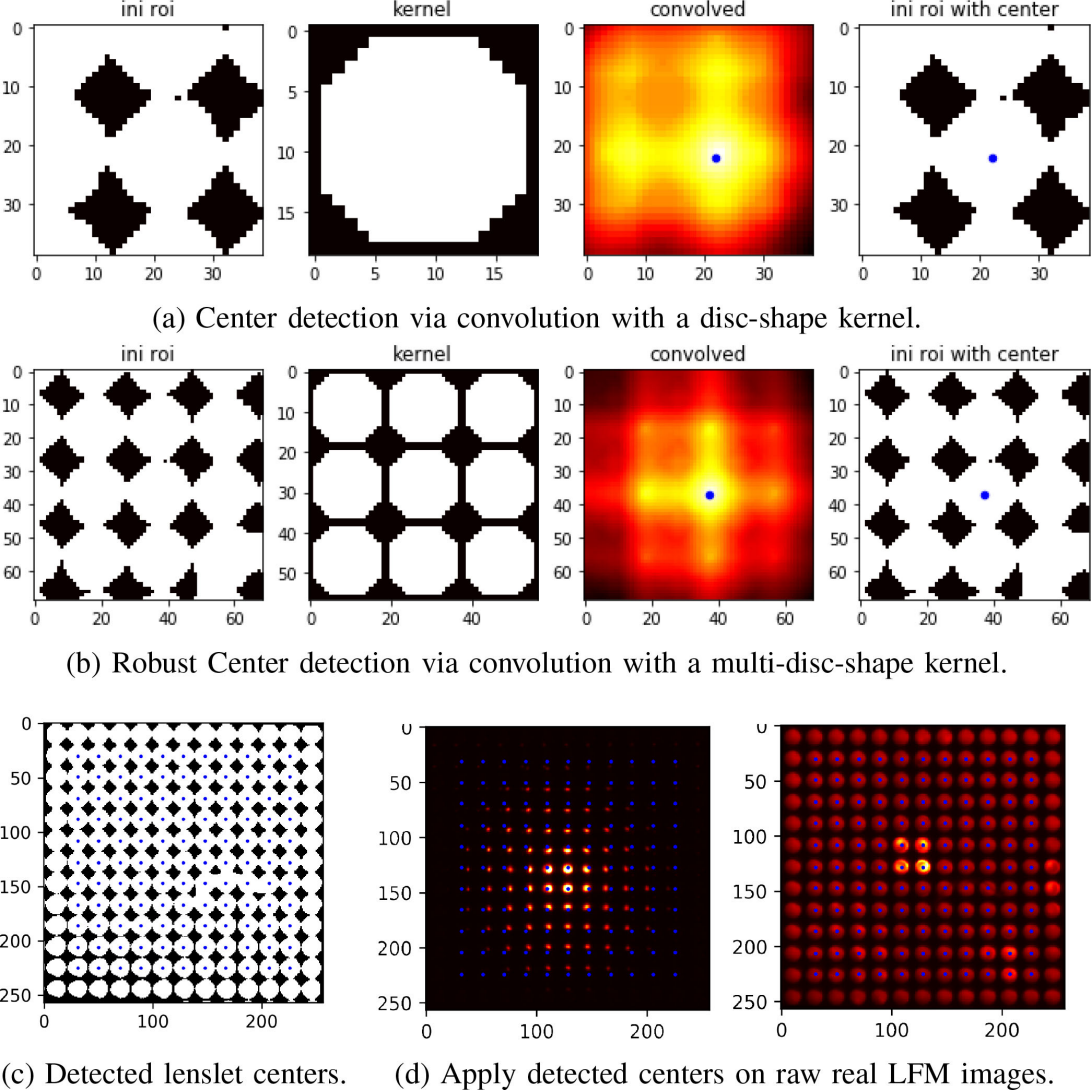
Automatic center detection via convolving the region of interest (ROI) with a designed kernel, such as (a) disc-shape kernel or (b) multi-disc-shape kernel. The latter kernel is more structured so that it exploits additional neighborhood information and makes the detection more robust. (c) Detect lenslet centers from a binarized out-of-focus light-field image. (d) Apply detected centers on raw real LFM images of a bead (left) and a neuron (right).

Given a LFM image with rectified rotation angle and detected lenslet pitch, the center detection is implemented based on the convolution of the LFM image with a properly designed kernel containing specific patterns, as described in Algorithm 1 and illustrated in Fig. 9(a)–(b).

Some center detection results are shown in Fig. 9(c)–(d). It is observed that the proposed method is able to robustly and accurately detect the centers of a MLA from an LFM image, even for those regions in the image where quality is poor. This is due to the convolution with a structured kernel which is designed to contain specific patterns so that it can take full advantage of neighbourhood information during the detection.

The obtained MLA centers and lenslet pitch from previous operations allow transforming a raw LFM image into the standard 4D format, further leading to EPI construction, as described in the next subsection.

#### 3) 4D LFM Data and EPIs

**Fig. 10.**
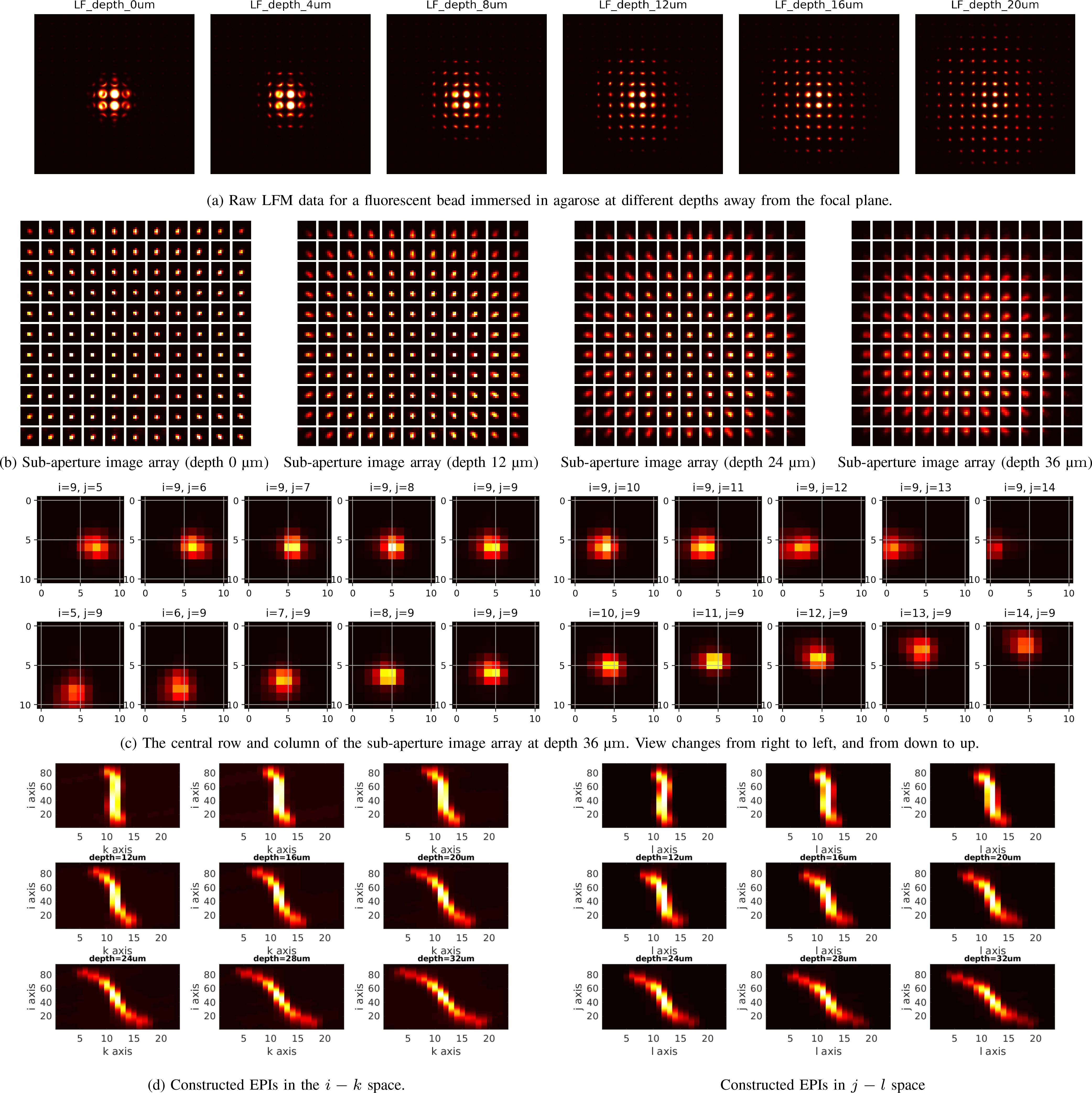
Non-scattering case. (a) Raw LFM images of a fluorescent bead immersed in agarose at different depths away from the focal plane. The pattern is expanded when the bead is far away from the focus plane. In each light-field image, we can see an array of small round spots which are the back-aperture of lenslets. The axes inside each spot are indexed by $i$ and $j$, while the positions of each spot are indexed by $k$ and $l$. (b) Sub-aperture image arrays for different depths. After a raw 2D light-field image is converted into the standard 4D format, we can re-arrange the pixels into sub-aperture images. Each sub-aperture image is composed of pixels that share the same relative position behind each lenslet. All the sub-aperture images can be tiled into an array with $k$-$l$ as the inside axes, and $i$-$j$ as the outside axes, just opposite as in the raw data. (c) From a row or a column of the sub-aperture image array, it is noticed that the positions of the bright area are shifting, which means the view direction of the bead and cell is changing horizontally or vertically. Such view changing accounts for the slope of epipolar lines in the EPIs. (d) Constructed $i-k$ and $j-l$ space EPIs. We concatenate a set sub-aperture images from a specified row (i.e. fixed $i$) or specified column (i.e. fixed $j$) of the sub-aperture image array in the third dimension. Then, each horizontal 2D slice (with fixed $k$) leads to an EPI in the $j-l$ space. Similarly, each vertical 2D slice (with fixed $l$) leads to an EPI in the $i-k$ space. Best seen by zooming on a computer screen.

**Fig. 11.**
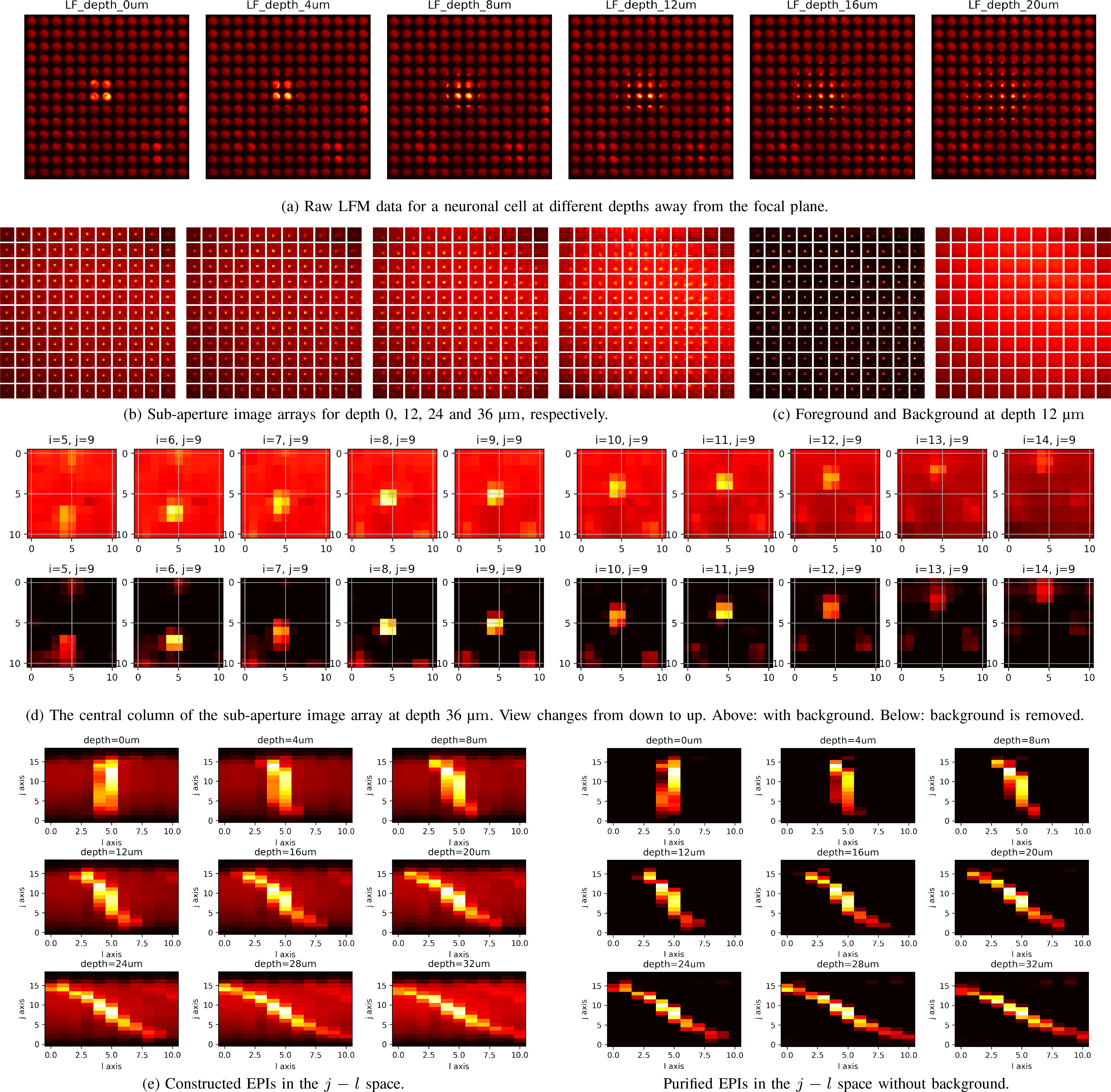
Scattering case. (a) Raw LFM images of a neuronal cell (from a genetically encoded mouse) at different depths away from the focal plane. The pattern is expanded when the neuronal cell is far away from the focus plane. Due to scattering and blurring, the cell images have a bright background and those for deeper positions have a weaker intensity contrast, thus making post-processing challenging. In each raw LFM image, we can see an array of small round spots which are the back-aperture of lenslets recorded in micro-images. (b) Sub-aperture image arrays for different depths. After a raw LFM image is converted into the standard 4D format, pixels can be re-arranged into sub-aperture images. Each sub-aperture image is composed of pixels that share the same relative position $(i,j)$ in behind each lenslet, indicating a specific view. All the sub-aperture images are tiled into an array with $k$-$l$ as the inside axes, and $i$-$j$ as the outside axes. (c) The separated foreground and background of a sub-aperture image array via matrix factorization. (d) From a column of the sub-aperture image array, it is noticed that the positions of the bright area are shifting, which means the view direction is changing vertically. Such view changing accounts for the slope of epipolar lines in the EPIs. The corresponding purified versions do not suffer from scattering as the background has been effectively removed by matrix factorization. (f) Constructed $j-l$ space EPIs and corresponding purified versions. Best seen by zooming on a computer screen.

Given the detected lenslet centers and pitch, we are able to extract each micro image, i.e. $I(:,:,k,l)$ for the $(k,l)$-th lenslet, from a raw 2D light-field image shown in Fig. 10(a) or 11(a), and then re-arrange them into a 4D matrix, thereby leading to the standard 4D format light-field $I_{4D}(i,j,k,l)$, ($\forall
                                i,j,k,l$). Once the raw 2D light-field image is decoded into the standard 4D format, sub-aperture images can be easily obtained by extracting 2D slices $I_{4D}(i,j,:,:)$ for specific views indexed by $(i,j)$. As aforementioned, each sub-aperture image is composed of pixels that share the same relative position behind each lenslet. All the sub-aperture images can be combined into an array with $(k,l)$ as the inside axes and $(i,j)$ as the outside axes, as shown in Fig. 10(b) and Fig. 11(b).

In each sub-aperture image array, each row indicates horizontal change of view (from right to left) while each column indicates vertical change of view (from down to up). This phenomenon becomes more apparent after extracting the central row and central column from a sub-aperture image array, as shown in Fig. 10(c) and Fig. 11(d). The view change accounts for the slope of epipolar lines in an EPI.

When constructing an EPI, a specified row (i.e. fixed $i$) of sub-aperture images are concatenated in the third dimension and then a 2D slice (for a fixed $k$) leads to an EPI in the $j -
                            l$ space. Similarly, a pair of fixed $j$ and $l$ leads to an EPI in the $i -
                            k$ space, as shown in Fig. 10(d) and Fig. 11(e). In these EPIs, one can easily notice aforementioned characteristics that the slope of an epipolar line is associated with the depth of a corresponding source – the deeper the source, the more tilted the epipolar line.

### B. 3D Localization

3D Localization is based on convolutional sparse coding on an observed EPI with respect to the synthesized EPI dictionary. Specifically, given an observed EPI (in vectorized form) $\mathbf {Y}\in
                            \mathbb {R}^{N}$ as input, we first solve a convolutional sparse coding problem (3):

\begin{align*} \underset{\mathbf {z}}{\min } \; & \frac{1}{2} \Vert
                            \mathbf {Y}- \sum\limits _{m=1}^{M} \mathbf {d}_m * \mathbf {z}_m \Vert
                            _2^2 + \beta \, \sum _{m=1}^{M} \Vert \mathbf {z}_m \Vert _1 \tag{3}
                            \end{align*}

where, $\mathbf {d}_m \in \mathbb {R}^{n} (n <
                            N)$ is the $m$-th atom of the EPI dictionary $\lbrace \mathbf
                            {d}_1,\ldots, \mathbf {d}_M\rbrace$ where each atom represents a vectorized EPI containing a single epipolar line associated with a specific depth. Moreover, $\mathbf {z}_m \in \mathbb
                        {R}^{N}$ is the corresponding coefficient map.

To solve Problem (3) efficiently, we transform the variables into the Fourier domain so that the convolutional operation in the original domain becomes element-wise multiplication, similar to [41]–[45]. Then, by exploiting the Parseval's theorem, we obtain:

\begin{align*}
                            \underset{\mathbf {z}_m}{\min } \; & \frac{1}{2} \Vert \hat{\mathbf
                            {Y}} - \sum _{m=1}^{M} \hat{\mathbf {d}}_m \odot \hat{\mathbf {z}}_m
                            \Vert _2^2 + \beta \, \sum _{m=1}^{M} \Vert \mathbf {z}_m \Vert _1
                            \tag{4} \end{align*}

where $\odot$ is element-wise product, i.e. Hadamard product, which corresponds to convolution in the original spatial domain. Here, $\hat{\mathbf {Y}} = \mathcal
                            {F} (\mathbf {Y}) \in \mathbb {R}^N$, $\hat{\mathbf {z}}_m =
                            \mathcal {F} (\mathbf {z}_m) \in \mathbb
                        {R}^N$, $\hat{\mathbf {d}}_m = \mathcal {F} (\mathbf {d}_m) \in
                            \mathbb {R}^N$, and $\mathcal {F}
                            (\cdot)$ indicates the Fourier transform operator.

To make the formulation concise, we define $\hat{\mathbf {D}} = [ \mathrm{diag} (\hat{\mathbf
                            {d}}_1),\ldots, \mathrm{diag}(\hat{\mathbf {d}}_M)] \in \mathbb {R}^{N
                            \times MN}$ as the whole dictionary, and $\mathbf {Z}= [\mathbf {z}_1;
                            \cdots ; \mathbf {z}_M]\in \mathbb {R}^{MN}$ as the concatenated version of all the column vectors $\mathbf {z}$. Then, Equation (4) becomes

\begin{align*}
                            \underset{\mathbf {Z}}{\min }\; & \frac{1}{2} \Vert \hat{\mathbf
                            {Y}} - \hat{\mathbf {D}} \hat{\mathbf {Z}} \Vert _2^2 + \beta \, \Vert
                            \mathbf {Z}\Vert _1 \,. \tag{5} \end{align*}

We use the alternating direction method of multipliers (ADMM) to solve the optimization problem in (5). Introducing auxiliary variable $\mathbf {T}$ leads to (6):

\begin{align*} \underset{\mathbf
                            {Z}}{\min } \quad& \frac{1}{2} \Vert \hat{\mathbf {Y}} -
                            \hat{\mathbf {D}} \hat{\mathbf {Z}} \Vert _2^2 + \beta \, \Vert \mathbf
                            {T}\Vert _1 \\ \text{s.t.} \quad& \mathbf {Z}= \mathbf {T}\tag{6}
                            \end{align*}

and the augmented Lagrangian of the objective is formulated as:

\begin{align*} \mathcal {L}(\mathbf {Z},\mathbf
                            {T},\boldsymbol{\gamma }) =& \frac{1}{2} \Vert \mathbf {Y}- \mathbf
                            {D}\mathbf {Z}\Vert _2^2 + \beta \Vert \mathbf {T}\Vert _1 +
                            \boldsymbol{\gamma }^\top (\mathbf {Z}-\mathbf {T})\\ &+ \frac{\mu
                            }{2} \Vert \mathbf {Z}- \mathbf {T}\Vert _2^2 \,. \tag{7} \end{align*}

Then, the following 3 subproblems are alternatively solved:

\begin{align*} \mathbf {Z}^{(i+1)} & = \arg \underset{\mathbf
                            {Z}}{\min } \; \mathcal {L}(\mathbf {Z},\mathbf
                            {T}^{(i)},\boldsymbol{\gamma }^{(i)}) \\ & = \arg \underset{\mathbf
                            {z}}{\min } \; \frac{1}{2} \Vert \hat{\mathbf {Y}} - \hat{\mathbf {D}}
                            \hat{\mathbf {Z}} \Vert _{2}^{2} + \boldsymbol{\gamma }^{(i)^\top }
                            (\hat{\mathbf {Z}}-\hat{\mathbf {T}}^{(i)})\\ &\quad + \frac{\mu
                            }{2} \Vert \hat{\mathbf {Z}} - \hat{\mathbf {T}}^{(i)} \Vert _{2}^{2} \\
                            & = \mathcal {F}^{-1} \lbrace (\hat{\mathbf {D}}^\top \hat{\mathbf
                            {D}} + \mu \mathbf {I})^{-1} (\hat{\mathbf {D}}^\top \hat{\mathbf {Y}} -
                            \hat{\boldsymbol{\gamma }} + \mu \hat{\mathbf {T}}^{(i)}) \rbrace
                            \tag{8} \\ \mathbf {T}^{(i+1)}& = \arg \underset{\mathbf {T}}{\min}
                            \; \mathcal{L}({\mathbf {Z}}^{(i+1)},{\mathbf {T}},\boldsymbol{\gamma
                            }^{(i)})\\ & =\arg \underset{\mathbf {t}}{\min} \; \beta \Vert
                            {\mathbf {T}} \Vert_1 + \boldsymbol{\gamma }^{(i)^\top} ({\mathbf
                            {Z}}^{(i+1)}-{\mathbf {T}})\\ &\quad + \frac{\mu}{2} \Vert {\mathbf
                            {Z}}^{(i+1)} - {\mathbf {T}} \Vert_{2}^{2}\\ &= {\mathbf
                            {S}}_{\beta/ \mu} ({\mathbf {Z}}^{(i+1)} + \boldsymbol{\gamma
                            }^{(i)}/\mu) \tag{9} \end{align*}

where $\mathbf {S}_{\beta /\mu }
                            (\cdot)$ is the soft-thresholding function defined as $S_\lambda (x) =
                            \text{sign}(x) \cdot (| x | - \lambda)_+$, and

\begin{align*}
                            \boldsymbol{\gamma }^{(i+1)} =& \arg \underset{\boldsymbol{\gamma
                            }}{\min } \; \mathcal {L}(\mathbf {Z}^{(i+1)},\mathbf
                            {T}^{(i+1)},\boldsymbol{\gamma }) = \boldsymbol{\gamma }^{(i)} \\ &+
                            \mu (\mathbf {Z}^{(i+1)} - \mathbf {T}^{(i+1)}) \,. \tag{10}
                            \end{align*}

To summarize, subproblem $\mathbf
                        {z}$ is a least square problem, subproblem $\mathbf
                        {t}$ is a $\ell _1$ regularized soft-thresholding problem, also called LASSO [46], and subproblem $\boldsymbol{\gamma }$ is to update the Lagrangian multipliers.

Once the coefficient maps $\lbrace
                            \mathbf {z}_1,\ldots, \mathbf {z}_M \rbrace$ are obtained, we compute a vector that contains the energy of each maps, and then the $S$ centroids $\lbrace \mathbf {z}_\Omega
                        \rbrace$ can be found via clustering on the energy vector, where $S$ denotes the number of point sources and $\Omega$ is the set containing the indices of the $S$ representative coefficient maps. Then, the target atoms $\lbrace \mathbf {d}_\Omega
                            \rbrace$ indexed by $\Omega$ lead to the depths directly, while the peak value in each selected coefficient map indicates the lateral positions. Together, they give the 3D positions $\lbrace \mathbf {p}^{(s)} \in \mathbb {R}^3, s = 1,\ldots, S
                            \rbrace$ of associated point sources.

However, in practice, due to blurring, noise and scattering, the coefficient maps $\mathbf
                        {Z}_h$ of convolutional sparse coding on horizontal EPIs (i.e. in $i-k$ space) may be different from $\mathbf
                        {Z}_v$ on vertical EPIs (i.e. in $j-l$ space). Therefore, the 3D location $\mathbf
                            {p}^{(s)}_h$ found using $\mathbf
                        {Z}_h$ are often different from the $\mathbf
                            {p}^{(s)}_v$ found using $\mathbf
                        {Z}_v$. To determine which one to select, a naive way is to average the two estimated locations. Alternatively, we suggest taking advantage of coefficient maps to compute weights which assess how well the convolutional sparse coding has performed on the EPIs and how reliable the estimated locations are. In this way, the weights act as auxiliary information to facilitate the manipulation of the results. In particular, considering that the target atoms should have a large response in corresponding coefficient maps, we compute a ratio $w$ (i.e. $w_h$ or $w_v$) for each coefficient matrix $\mathbf
                        {Z}$ (i.e. $\mathbf {Z}_h$ or $\mathbf
                        {Z}_v$) as the weights using:

\begin{equation*} w = \Vert \mathbf
                            {Z}_{:,\Omega } \Vert _F^2 / \Vert \mathbf {Z}\Vert _F^2. \tag{11}
                            \end{equation*}

Given the weights $w_h,
                        w_v$ and 3D locations $\mathbf {p}_h, \mathbf
                        {p}_v$, the final locations are obtained via weighted averaging:

\begin{equation*} \mathbf {p}^{(s)} = w_h \mathbf {p}^{(s)}_h + w_v
                            \mathbf {p}^{(s)}_v. \tag{12} \end{equation*}

The overall localization process is summarized in Algorithm 2.

Algorithm 2: **Location Detection Algorithm.**
**Input:**
  Observed horizontal EPI $\mathbf {Y}_h$ and vertical EPI $\mathbf
                                    {Y}_v$;
  A pre-simulated EPI dictionary $\mathbf {D}$.
**Output:**
  Coefficient maps $\mathbf
                                    {Z}_h$ and $\mathbf {Z}_v$; 3D locations of sources $\mathbf
                                    {p}^{(1)}, \ldots, \mathbf
                                {p}^{(s)}$.
**Procedures:**
1) **Convolutional Sparse Coding**
  Solve Convolutional Sparse Coding problem (6) via alternating between (8), (9) and (10) to obtain $\mathbf
                                    {Z}_h$ and $\mathbf
                            {Z}_v$.
2) **Detecting 3D locations**
  From $\mathbf
                                    {Z}_h$ and $\mathbf {Z}_v$), find the indices $\Omega$ of the $S$ representative coefficient maps, which lead to $\mathbf
                                    {p}^{(s)}_h$ (resp. $\mathbf
                                    {p}^{(s)}_v$).
3) **Computing weights**
  Computing weights for each EPI using (11).
4) **Computing final 3D locations**
  Perform weighted average using (12) to get final 3D locations.

## V. Experiments

In this section, we evaluate the 3D localization capabilities of the proposed method. We also compare our approach with the 3D deconvolution-based method (3D-Deconv for short) [24] where 3D deconvolution is followed by 3D localization, and the phase-space based method (Phase-Space for short) [28], [30] on both non-scattering and scattering specimens. In particular, for the case without scattering, we image a suspension of fluorescent beads of 10 $\mu {\text
                        m}$ diameter in agarose to get the raw LFM data, as shown in Fig. 10(a). For the case involving scattering, we use mouse brain tissues as specimens, and the obtained raw light-field data is shown in Fig. 11(a).

The light-field images of specimens were captured by systematically changing the distance between the objective lens and the specimens. Therefore, each light-field image corresponds to a specified depth and captures a 3D volume, not a single layer. In other words, each light-field image is an observation of the whole 3D space, rather than a slice of it. Furthermore, each light-field image is used independently to detect 3D positions of sources. Note that, 3D-Deconv [24] exploits a layer-by-layer approach to reconstruct the whole 3D volume using the Richardson-Lucy deconvolution algorithm. However, such layer-by-layer reconstruction is completely unrelated to our approach and the Phase-Space method [28], [30] as they focus on localization, i.e. detecting 3D positions of sources, instead of 3D volume reconstruction. This is a significant difference from 3D-Deconv [24]. All the localization experiments were conducted using MATLAB R2018a in a computer equipped with an Intel hexa-core i7-8700 U CPU at 3.20 GHz with 28 GB of memory, and 64-bit Ubuntu operating system.

### A. Experimental Setup and Data Preprocessing

We provide a brief introduction to the experimental setup to describe how the light-field data is acquired and preprocessed. More details about the experimental setup can be found in Section VII-B in the supplemental material.

*Non-scattering case:* The non-scattering samples used in our experiment are static suspension ($5.0*10^3 \mu L^{-1}$) of fluorescent beads with 10 $\mu {\text
                            m}$ diameter and sparsely distributed in a slice of agarose gel. To obtain ground-truth positions of the beads, a wide-field microscope (the same as the LFM but with the MLA removed) is used to scan the imaging volume at a sequence of depths. This leads to an image stack where each image frame corresponds to a specific depth. By changing the depth gradually, the depth and spatial positions for each target bead can be manually found when it is in focus at a specific image frame. A set of single-shot light-field frames are obtained for a bead at different depths 0 - 48 $\mu {\text
                            m}$ away from the focal plane. Some examples of the raw light-field images for fluorescent bead immersed in non-scattering media are shown in Fig. 10(a). Obviously, the light-field pattern recorded by the sensor is expanded when the bead is further away from the focus plane.

Given measured 2D raw LFM data, we perform calibration, and then convert the data into the two-plane parameterized 4D format followed by the construction of EPIs using the procedures introduced in Section IV-A. Fig. 10(b)–(c) show the sub-aperture images converted from the raw light-field images. Fig. 10(d) shows the constructed EPIs. Evidently, the bead forms a tilted epipolar line in the EPI with the slope inversely proportional to its depth.

*Scattering case:* The scattering samples used in our experiment are from brain tissues of a genetically encoded mouse. The imaging, calibration and decoding procedures are similar to that for fluorescent beads. However, scattering tissues induce blurs and background noise in the light-field images and consequently in the EPIs constructed from them, as shown in Fig. 11(a)–(b). Point sources located at deeper positions suffer more from blurring and noise. Such corruption may hinder the localization operation and result in performance degradation.

To alleviate the interference, we develop a set of purification operations. First, we vectorize all the sub-aperture images into column vectors and concatenate them into a 2D matrix $A$. We then perform a singular value decomposition (SVD) based matrix factorization operation on the matrix $A$ to get the largest singular value $\sigma
                        _{max}$ and corresponding singular vectors $u_{max}$ and $v_{max}$ so that the rank-one matrix $B = u_{max} \sigma
                            _{max} v_{max}^\top$ represents the background. Subsequently, the foreground can be separated out by subtracting the background $B$ from $A$, followed by re-arranging each column vector back into a 2D image, as shown in Fig. 11(c)–(d). Then, we construct EPIs using max-projection^1^^1^Given a 2D EPI image $X$ with $i = 1, \ldots, I$, $j = 1, \ldots,
                                    J$ representing row and column indices, respectively, the max-projection onto the vertical axis implies picking, from each row $X_{i,:}$, the brightest pixel $X_{i,max}$ and concatenating them as a vector $[X_{1,max},
                                    \ldots, X_{i,max}, \ldots,
                                X_{I,max}]$. Similarly, max-projection onto the horizontal axis implies picking, from each column $X_{:,j}$, the brightest pixel $X_{max,j}$ and concatenating them as a vector $[X_{max,1},
                                    \ldots, X_{max,j}, \ldots,
                                X_{max,J}]$. which projects the largest value per row and per column onto the vertical and horizontal axes, respectively. This also helps to reduce the interference of background blurring. Alternatively, referring to the spatial positions detected from the center-view sub-aperture image, one can also extract EPIs at those specified positions. This may lead to EPIs containing very few epipolar lines and therefore benefits the subsequent localization procedure. The remaining noise in constructed EPI can be further attenuated via using some denoising techniques. However, we found that such denoising operation is optional, as the capabilities of the proposed method are not significantly affected by the presence of interference due to robustness induced by sparsity.

### B. Experimental Results and Discussion

Given the constructed EPIs, 3D localization is achieved using proposed Algorithm 2 to perform convolutional sparse coding with respect to a synthetic EPI dictionary. The dictionary elements found for sparse representations indicate the depth positions while the obtained coefficients a.k.a feature maps lead to transverse positions, with reconstructed clean EPIs as by-product. The estimated 3D locations are compared with the proxy of groundtruth to obtain Root Mean Square Error (RMSE) for evaluating the performance.

**Fig. 12.**
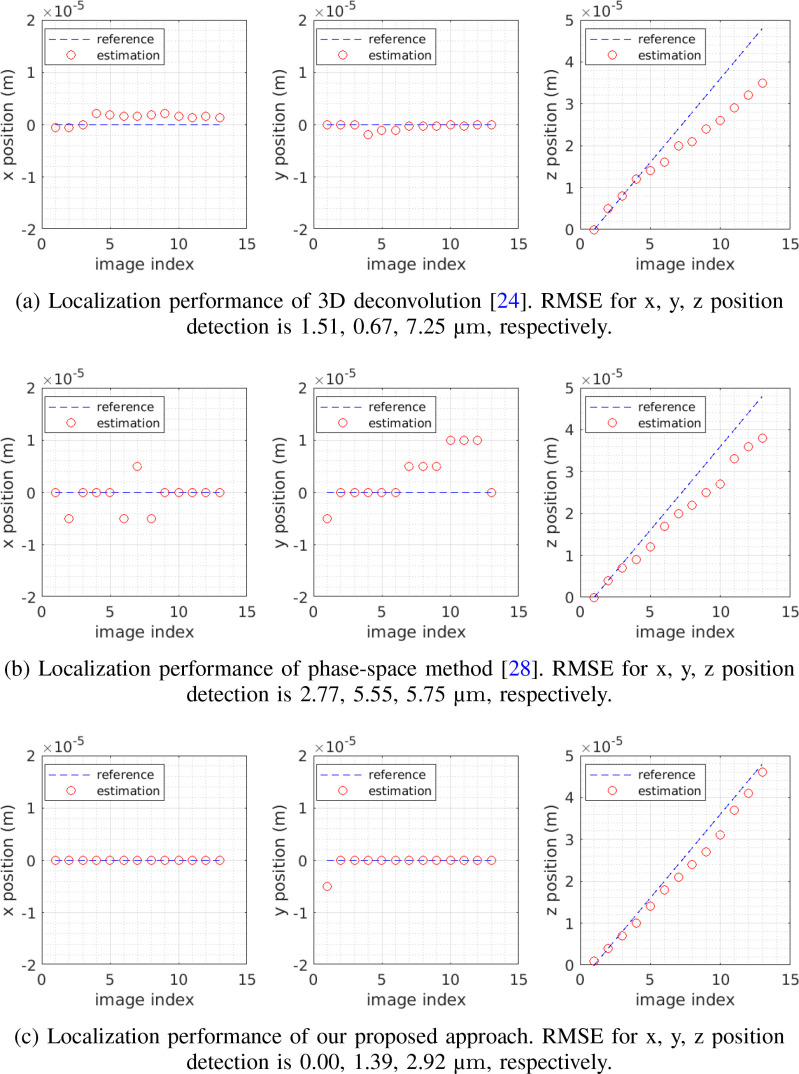
Non-scattering case. Compare performance of localizing a fluorescent bead using three different methods, including the 3D deconvolution [24], phase-space method [28] and our proposed method. Depth varies from 0 $\mu {\text m}$ to 48 $\mu {\text
                                    m}$.

**Fig. 13.**
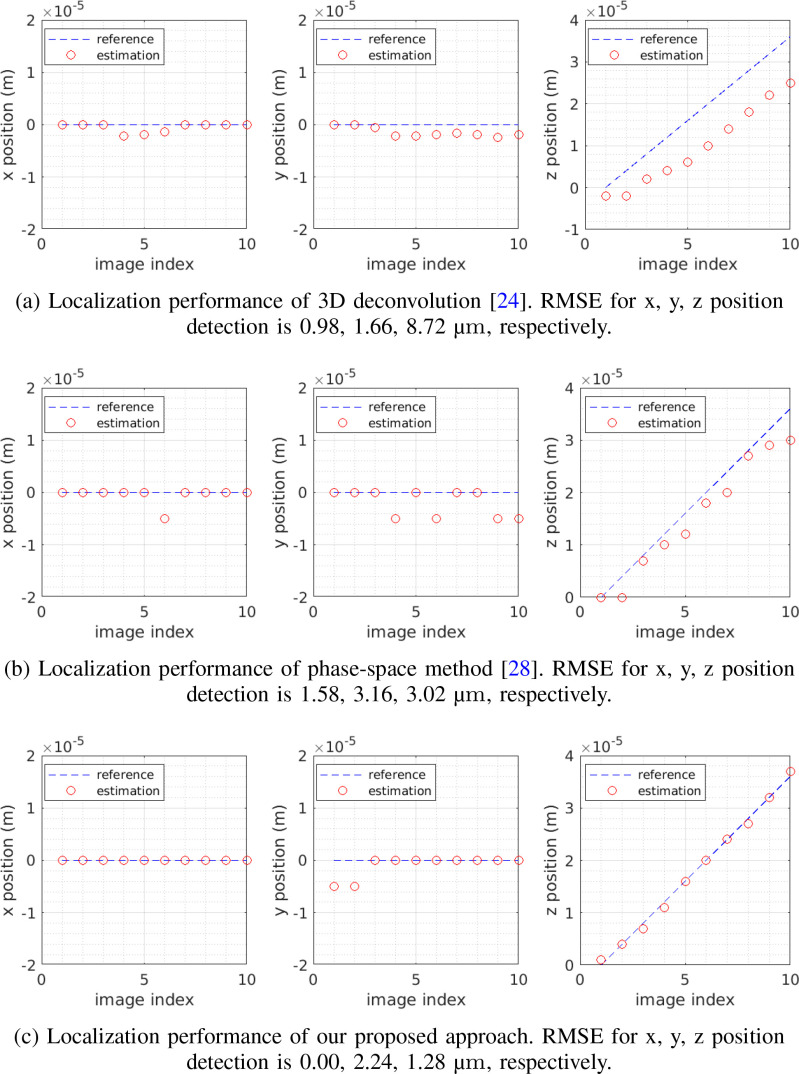
Scattering case. Compare performance of localizing a neuronal cell using three different methods, including the 3D deconvolution [24], phase-space method [28] and our proposed method. Depth varies from 0 $\mu {\text m}$ to 36 $\mu {\text
                                    m}$.

The results for non-scattering and scattering cases are shown in Fig. 12 and Fig. 13, respectively. In general, 3D-Deconv [24] and our approach perform well on localizing transverse positions, i.e. x and y coordinates, and both outperform Phase-Space [28], [30]. However, when it comes to detecting depth positions, our approach and Phase-Space [28], [30] demonstrate superior performance and outperform 3D-Deconv [24] with notable gains. We also note that the performance of both 3D-Deconv [24] and Phase-Space [28], [30] suffer more at increased depths than our approach.

**Fig. 14.**
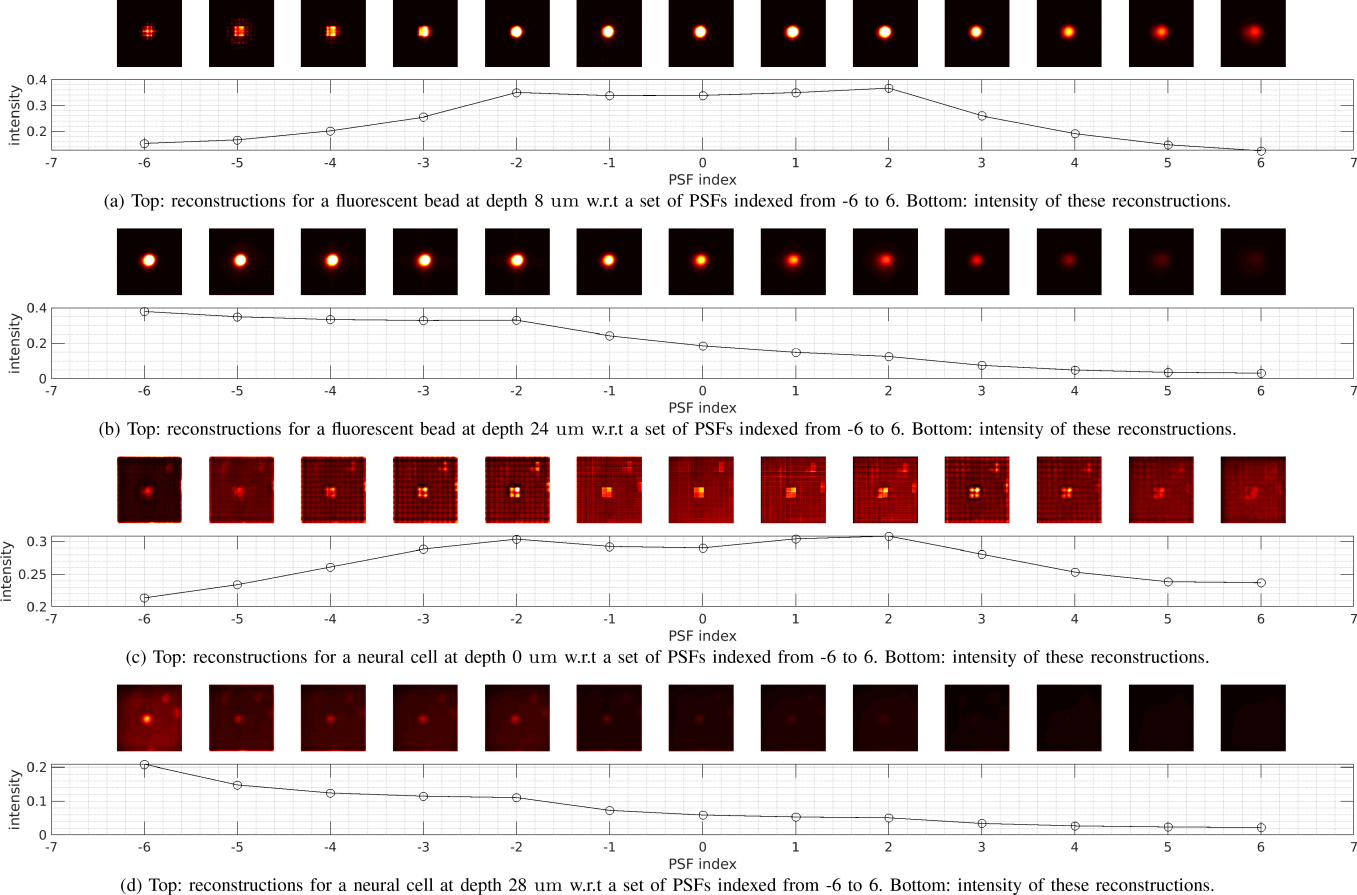
3D deconvolution [24] tends to give large deviation in depth detection. In each subfigure, the first row denotes reconstructed 2D images from light-field of a fluorescent bead and a neural cell via 3D deconvolution with respect to (w.r.t) a set of PSFs that correspond to a set of different depths. In each subfigure, the middle image represents the reconstruction w.r.t the correct PSF (indexed by 0). The images on the left side represent reconstructions w.r.t shallower PSFs (indexed by a negative number), while the images on the right side represent reconstructions using deeper PSFs (indexed by a positive number). It is evident that when the source is at a shallow position, e.g. 8 $\mu {\text m}$ for a fluorescent bead, the correct PSF and its adjacent PSFs give the best reconstruction, leading to correct depth detection. However, for the source at a deep position, e.g. 24 $\mu {\text m}$ or beyond, reconstructions w.r.t shallower PSFs are better than that w.r.t the correct PSF, leading to increased underestimation errors.

The good performance of 3D-Deconv [24] on localizing transverse positions is mainly due to the super-resolution effect induced by Richardson-Lucy deconvolution algorithm. This effect results in a finer discretization ($\delta = \frac{d}{M*N_i} = \frac{125}{25 \times 19} =
                            \text{0.26}\;\mu {\text m}$) in x and y coordinates, even smaller than the resolution limit ($\delta = \frac{d}{M} = \text{5}\;\mu {\text
                            m}$) of light-field microscopy, where $d$ is lenslet pitch, $M$ is the magnification factor, and $N_i$ is the number of pixels behind each lenslet. On the other hand, 3D-Deconv [24] suffers from significant degradation of performance on detecting depth positions. This is because, for a deep position, both the correct PSF (2D matrix) and similar PSFs may produce similar reconstructions, and what is more problematic is the fact that the PSFs corresponding to shallower depths give better reconstructions with higher intensity than the correct PSF. As shown in Fig. 14, it can be seen that the reconstructions from a range of PSFs can be similar, and better reconstructions with higher intensity tend to be obtained with respect to shallower PSFs, rather than the correct PSF. This is why it experiences an increased underestimation error with increasing depth. Such performance degradation becomes even more severe for a scattering case, as shown in the rightmost figures 12(a) and 13(a).

**Fig. 15.**
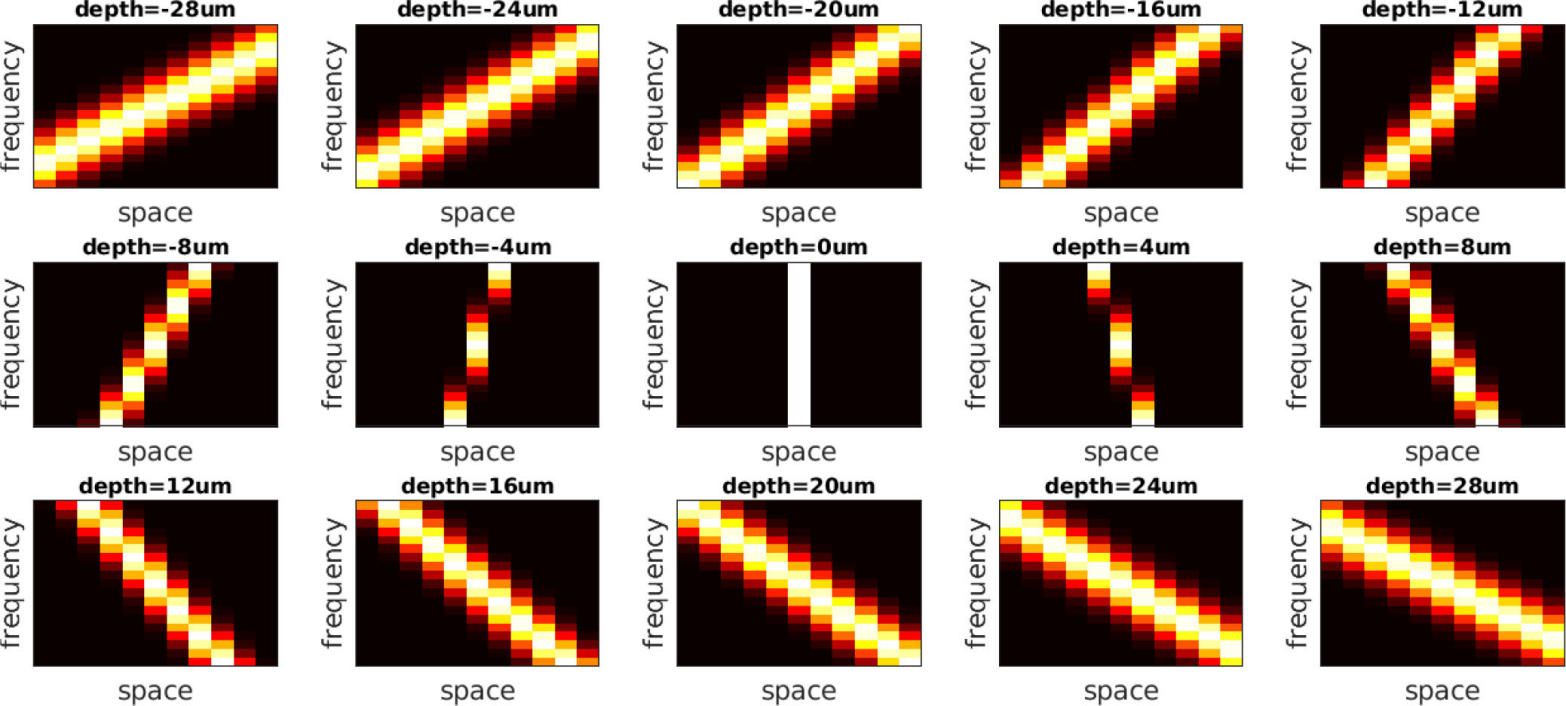
Phase-space dictionary model [28], [30]. It is noticed that the phase-space dictionary elements are straight lines with uniform shearing everywhere. There exists notable mismatches between the phase-space dictionary and real light-field observations, which cause localization errors, in particular at deeper positions.

**Fig. 16.**
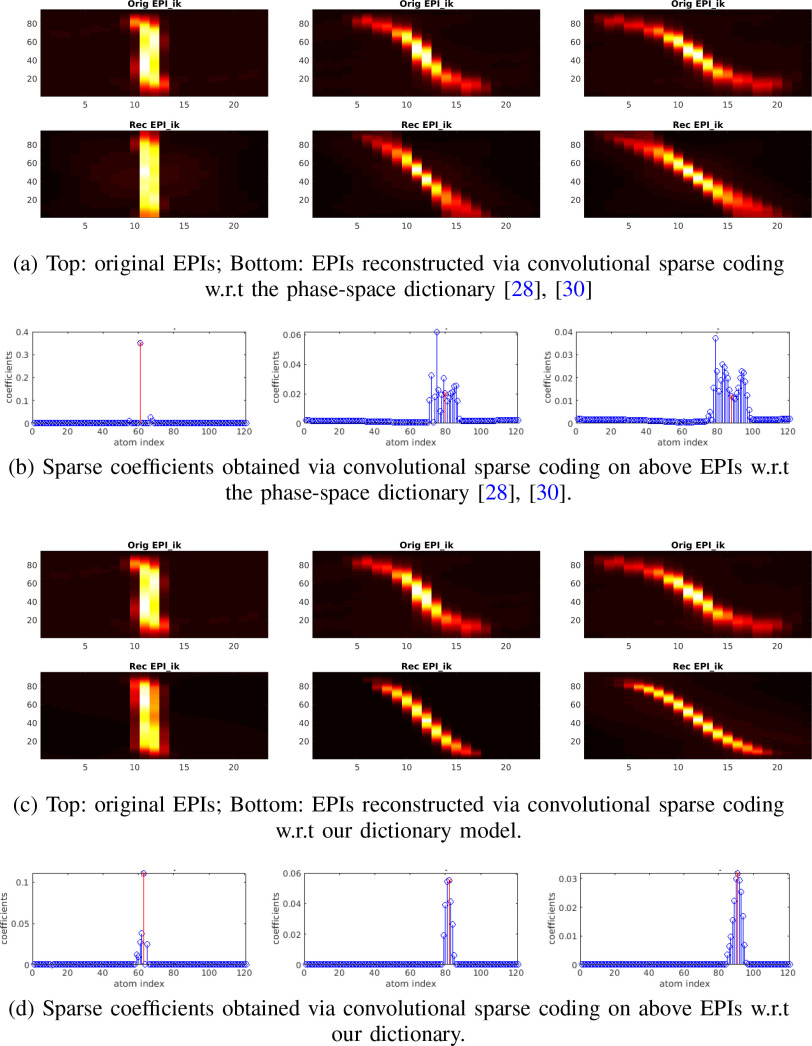
Due to mismatch between the synthesized phase-space dictionary and the real light-field observation, the reconstructed EPIs with respect to the phase-space dictionary model [28], [30] can not represent original EPIs well enough, as shown in (a), and the sparse coefficients degrade in particular for deeper sources, as shown in (b). The reconstruction and sparse coefficients, as shown in (c) and (d) with respect to our dictionary model demonstrate better quality and structured sparsity than  [28], [30].

Phase-Space [28], [30] incorporates the wave-optical and geometric effects into their model using a phase-space Wigner function (and its Fourier spectrogram) so that the light propagation in space can be easily represented by a simple shearing operation in phase-space. However, the effects of the main lens and the microlens array were ignored. The fact that these effects were not fully incorporated may account for why their phase-space dictionary elements are straight lines with uniform shearing everywhere, as shown in Fig. 15. It can be noticed that the simulated dictionary elements do not resemble real phase-space observations, in particular at deeper positions, as shown in Fig. 10(d) and Fig. 11(e), where the real observations exhibit an ‘S’-shape due to distortion and aberrations from the lenses. In addition, the elements in the phase-space dictionary [28], [30] are PSFs that correspond to ideal point sources without considering a reasonable radius. All these mismatches may reduce robustness, leading to notable localization errors, in particular at deeper positions, as shown in figures in Fig. 12(b) and Fig. 13(b). Note that, for a fair comparison, we have applied the same convolutional sparse coding algorithm for both Phase-Space [28], [30] and our approach, and the only difference is in the design of the dictionary. We also test a set of scattering parameters and select the best one for Phase-Space [28], [30]. As shown in Fig. 16, the coefficients are sparse and the reconstructions are of good quality for shallower positions, but they degrade considerably for deeper positions as straight lines in the phase-space dictionary are not able to represent the ‘S’-shape observations well enough. In spite of these drawbacks, Phase-Space [28], [30] still outperforms 3D-Deconv [24] on depth detection with significant improvements, in particular for scattering cases, owing to more structured patterns and redundant information of light-fields in phase-space.

The enhanced localization performance of our approach is due to the accurate light-field model and EPI dictionary, as well as convolutional sparse coding-based localization algorithm. In addition to wave-optical effects, our light-field model also considers the effect of the main lens and the microlens array of the microscopy system along the whole light-field propagation path, which ensures our model represents the real observations more accurately than the model [28], [30]. In particular, the main lens together with the relevant 4F system makes sure that the electromagnetic field is approximately band-limited in space. They also result in non-uniform light distribution in the imaging plane so that the light density is the largest at the center of the imaging plane and becomes smaller for areas far away from the center. This accounts for why epipolar lines in our EPIs tend to be thicker at the center region and thinner at the two ends, as shown in Fig. 6, in particular for out-of-focus sources, e.g. at a depth of 20 um. This phenomenon also matches real light-field observations, as shown in Fig. 10(d) and Fig. 11(e). In addition, the blurring and downsampling effect from the microlens array and associated pixels behind each lenslet are also incorporated into the model. Owing to the exploitation of EPI/phase-space, our approach exhibit a similar advantage to Phase-Space [28], [30] in detecting depth positions over 3D-Deconv [24]. Furthermore, the more accurate dictionary model contributes to enhanced sparsity and robustness because fewer atoms are required for good representation. This enables us to outperform Phase-Space [28], [30] at detecting deeper positions as well as transverse positions. In addition, even though our approach also focuses on localization without super-resolution effects in the transverse dimension, thus preventing improvements to discretization in x and y coordinates, we still obtain competitive transverse localization performance with comparable RMSE to 3D-Deconv [24]. This is because our approach improves localization accuracy and thereby counteracts the adverse impact of discretization to some extent. To summarize, based on the accurate model and effective algorithm, our approach demonstrates higher localization accuracy and robustness than previous methods, as shown in Fig. 12(c) and Fig. 13(c). It produces the best 3D localization performance at depth in scattering conditions that normally prevent good localization in particular along the axial (z) dimension.

We refer to the supplemental material (Subsection VII.B and VII.C) for further results and for further discussion.

## VI. Conclusion

A single LFM image captures 4D geometrics of light rays, including both spatial and angular information. We propose an efficient 3D localization approach to detect 3D positions of neuronal cells from a single light-field snapshot. Our approach first calibrates and decodes the raw light-field image into the standard 4D format and then construct EPIs. By leveraging EPIs as effective features, we perform convolutional sparse coding with respect to a depth-aware synthesized EPI dictionary to achieve 3D localization of targets. Since the proposed approach skips time-consuming and error-prone 3D volume reconstruction, it improves the efficiency and accuracy of the 3D localization. Experiments on both non-scattering and scattering media demonstrate that our approach can reliably detect the 3D positions of granular targets with high fidelity and also exhibits outstanding robustness to scattering and aberration effects.

## Supplementary Material

10.21227/864p-p592 Light-field microscopy data

This article has supplementary downloadable material available at https://ieeexplore.ieee.org, provided by the authors.
